# Outer membrane changes enable evolutionary escape from bacterial predation

**DOI:** 10.1093/ismejo/wrag235

**Published:** 2026-09-16

**Authors:** Subham Mridha, Ove Christopher Mattmann, Sarah-Luise Loose, Saskia Isele, Bernd Roschitzki, Rolf Kümmerli, Simona G Huwiler

**Affiliations:** Department of Plant and Microbial Biology, University of Zurich, Zurich CH-8008, Switzerland; Department of Quantitative Biomedicine, University of Zurich, Zurich CH-8057, Switzerland; Department of Plant and Microbial Biology, University of Zurich, Zurich CH-8008, Switzerland; Department of Quantitative Biomedicine, University of Zurich, Zurich CH-8057, Switzerland; Department of Plant and Microbial Biology, University of Zurich, Zurich CH-8008, Switzerland; Department of Plant and Microbial Biology, University of Zurich, Zurich CH-8008, Switzerland; Functional Genomics Center Zurich, University of Zurich/ETH Zurich, Zurich CH-8057, Switzerland; Department of Quantitative Biomedicine, University of Zurich, Zurich CH-8057, Switzerland; Department of Plant and Microbial Biology, University of Zurich, Zurich CH-8008, Switzerland

**Keywords:** predation resistance, antimicrobial resistance, experimental evolution, predator–prey interactions, predatory bacteria, *Bdellovibrio bacteriovorus*, proteomics, genomics, *Escherichia coli*, living antibiotics

## Abstract

Antimicrobial resistance (AMR) is a threat to modern medicine. To combat AMR pathogens, natural predators like bacteriophages and predatory bacteria have gained interest recently. Predatory bacterium *Bdellovibrio bacteriovorus* is ubiquitous and has a broad prey range. It is particularly potent at killing many AMR Gram-negative bacterial pathogens featured on the WHO priority list. However, it is currently unclear whether prey bacteria can evolve genetically determined resistance against predation by *B. bacteriovorus*. Here, we show that the model bacterium *Escherichia coli* K-12 consistently evolves resistance against *B. bacteriovorus* during experimental evolution. Selection for resistance scaled positively with predation pressure and was widespread after two cycles of predator exposure. Similar to antibiotics, predation resistance was costly, manifesting in a trade-off between predation resistance and fitness in the absence of predators. Genetic analysis combined with proteomics identified mutations that lead to the down-regulation of the outer membrane porin OmpF as a common resistance mechanism. In addition, a rarer mutation in cell envelope lipopolysaccharide-modifying enzyme WaaF also conferred predation resistance, likely by a pleiotropic effect, which included OmpF down regulation. While our study uncovers evolutionary and mechanistic aspects of prey escape from predation, it also highlights that the high cost of resistance reflects a handicap for the pathogen and can thus be exploited to increase treatment sustainability. Altogether, our work generates essential knowledge in ecologically important predator–prey interactions and can advance predatory bacteria as “living antibiotics” to combat AMR.

## Introduction

Bacterial resistance to conventional antibiotics is a major public health concern, which led to 1.27 million deaths in 2019 [1]. This urgently calls for the discovery of novel antibiotics and alternative treatment strategies [2–4]. One promising alternative involves the use of natural bacterial predators including bacteriophages (viruses of bacteria) [5] and predatory bacteria [3, 6], which are environmentally widespread [7]. While phage therapy is currently extensively studied [2], much less is known about the potential of predatory bacteria, like *Bdellovibrio bacteriovorus*, that kill and invade pathogenic bacteria and are therefore considered living antibiotics [3, 6]. *Bdellovibrio bacteriovorus* is capable of killing growing and stationary phase bacterial prey cells, as well as antibiotic resistant strains [8–10].

Similar to bacteriophages, *B. bacteriovorus* is neither toxic nor pathogenic to eukaryotic cells and is therefore of great promise [3, 6]. However, key differences exist between the two predator types. For example, bacteriophages are typically very specific to prey strains, whereas *B. bacteriovorus* is a generalist with a broad prey range [11, 12]. This makes *B. bacteriovorus* suitable to treat polymicrobial infections [11, 13]. Moreover, there seem to be few defense and immune mechanisms present among prey species to defend themselves against *B. bacteriovorus* [14, 15]. This contrasts with the over hundred specific bacterial defense and immune systems reported against bacteriophages [16]. Thus, *B. bacteriovorus* might be a more effective treatment under certain circumstances, compared to traditional antibiotics and phage therapy.

Despite these promising aspects, little is known on the selection pressures that *B. bacteriovorus* may impose on its prey and the evolutionary responses it may trigger [17–19]. Some evolutionary studies, focusing on predator–prey interactions, reported evidence for resistance evolution [17–19] but did not explore the underlying mechanisms in detail. Although recognition of the prey and prey invasion is likely multifactorial [20, 21], potentially hindering fast resistance formation, prey resistance has often been described transient [3, 22]. One study described a form of phenotypic (plastic) resistance, whereby several prey species developed a transient yet reversible stage of predation resilience [22]. Further, several studies highlighted that secretion systems [23] and secreted compounds such as quorum-sensing signals [23, 24], indole [25] and cyanide [26] may offer some protection from *B. bacterivorous* predation, while the presence of paracrystalline protein surface (S-) layers seems to prevent killing in certain bacterial species [14].

To obtain a systematic and in-depth understanding of the potential of prey to evolve resistance to *B. bacteriovorus*, we performed an experimental prey evolution study with the model organism *Escherichia coli* K-12 as prey and *B. bacteriovorus* HD100 as predator. Upon repeated and intermittent exposure of prey to different predator concentrations, we found strong evidence that *E. coli* K-12 predominantly evolves genetically based predation resistance under high predatory pressure, that occurred repeatedly across independently evolved prey lineages. Predation resistance is costly and manifested in a negative association between the resistance level of a lineage and its growth in the absence of predation. A combination of population sequencing at high genome coverage, proteomics and single-gene deletion mutant experiments revealed that mutations in the outer membrane porin OmpF gene, and its downregulation are primarily involved in predation resistance. Moreover, a mutation in enzyme WaaF gene involved in lipopolysaccharide (LPS) modification also exhibited a resistant phenotype. These results suggest that prey evolution can lead to modifications of outer membrane components, affecting different potential points of contact with the predator [15, 20]. Our study elucidates the ecological context, the evolutionary path, and the molecular mechanisms of prey resistance evolution against *B. bacteriovorus*, generating key knowledge for the development of bacterial predators as sustainable living antibiotics.

## Materials and methods

### Growth of bacterial strains

In this study, *E. coli* K-12 MG1655 (DSM 18039) as prey and *B. bacteriovorus* HD100^T^ as predator were used, unless indicated otherwise. *B. bacteriovorus* HD100^T^ was obtained as a kind gift from the laboratory of Prof. R. E. Sockett at University of Nottingham, UK. *B. bacteriovorus* HD100 was cultured and maintained on Ca/HEPES buffer (25 mM HEPES, 2 mM CaCl_2_, pH 7.6) together with late-log phase *E. coli* K-12 MG1655 as prey, at 29°C for 24 h at 200 rpm shaking [27]. Revival of *B. bacteriovorus* and determination of plaque forming units (PFU) for enumeration was done according to protocols by Lambert & Sockett [27] and is described in detail in the Supplementary Methods and Materials.

All *E. coli* strains used are listed in Supplementary Table S1. To validate the genetic effect on predation resistance, markerless single deletion strains Δ*envZ,* Δ*ompF,* Δ*truA,* Δ*waaB,* and Δ*waaF* were generated (details in Supplementary Methods and Materials, primer details in Supplementary Table S2) based on *E. coli* KEIO library strains [28] containing an inserted kanamycin resistance cassette. The different *E. coli* strains were grown in YT broth (Yeast tryptone, 5 g l^−1^ NaCl, 5 g l^−1^ Bacto yeast extract, 8 g l^−1^ tryptone, pH 7.5) at 37°C with 200 rpm shaking overnight, and additional appropriate antibiotics as indicated. For ancestor prey, *E. coli* K-12 MG1655 was retrieved from 20% glycerol stocks at −80°C and were streaked on a YT agar plate (YT broth with 10 g l^−1^ agar) and incubated at 37°C for 24 h to obtain single colonies. The overnight cultures from ancestor prey single colonies were used for 24-h lysates (growth of predator), revival and enumeration of the predator in our experiments.

### Experimental prey evolution

In the experimental prey evolution, *E. coli* K-12 MG1655 was used as ancestral prey strain and subjected to three different predatory pressures by *B. bacteriovorus* HD100 involving 15 alternate cycles of predation and recovery phase (Fig. 1a). The detailed protocol is described in the Supplementary Materials and Methods. The predation phase occurred in rich medium (containing three-fourths YT, one-forth 25 mM HEPES and 2 mM CaCl_2_) allowing growth of *E. coli* K-12 as well as predation of *B. bacteriovorus* at 200 rpm and 29°C. *E. coli* K-12 prey were subjected to three different predatory conditions in eight-fold independent replication each, that resulted in 24 distinct lineages: No predatory exposure, low predatory exposure (multiplicity of infection [MOI], ratio of predator (PFU/ml) vs prey cells (CFU/ml), was ~2.5) and high predatory exposure (MOI ~10). The three conditions were chosen to exert no, low and high selection pressure on the prey, respectively. After the 24-h predation phase surviving *E. coli* were separated from *B. bacteriovorus* by filtration to avoid co-evolution. In the subsequent 16-h recovery phase retrieved *E. coli* from filtration were diluted ~1:100 and grown in YT broth at 200 rpm and 37°C. These overnight cultures were diluted back to an overall initial OD_600nm_ of 0.003 for the next predation phase and evolution cycle.

**Figure 1:**
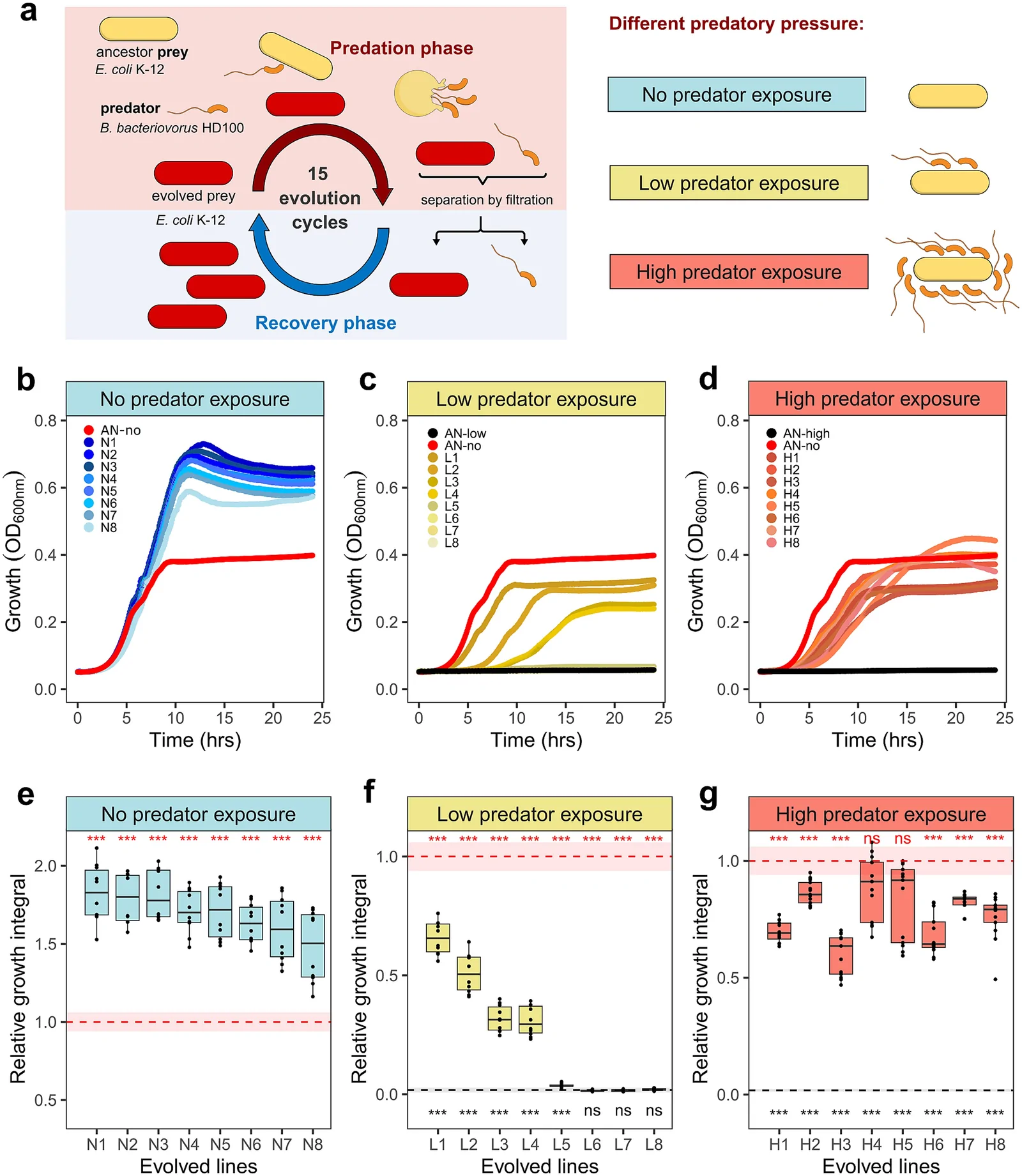
Repeated exposure of predator *B. bacteriovorus* HD100 leads to growth of prey *E. coli* K-12 under predation. (a) Experimental evolution scheme: eight different prey lineages are exposed repeatedly to different concentration of the predator in the predation phase in a medium allowing for prey growth and predation: no (N1–N8, blue), low (L1–L8, yellow) and high (H1–H8, red) predatory pressure. Each prey evolution cycle is composed of an alternating predation phase (with no, low and high predator concentrations) and recovery phase (without predator), whereby surviving prey is separated after the predatory phase. Growth dynamics of evolved prey lineages after 15 evolution cycles: (b–d) growth curves measured as OD_600nm_ over time of eight independently evolved prey lineages under no (b), low (c) and high (d) predatory pressure when exposed to respective predation conditions where they evolved. They were compared with ancestor under no (AN-no) or specific predatory pressures (AN-low & AN-high). Each point of the growth curves represents average OD_600nm_ at that timepoint across replicates. (e-g) Relative growth integral of b–d, defined as growth integral (area under the curve) divided by the growth integral of ancestor under no predatory pressure. Boxplots represent the median with 25th and 75th percentiles, and whiskers show the 1.5 interquartile range. Red dotted line and shadow indicate the mean and standard deviation range of AN-no. Black dotted lines and shaded areas indicate the mean and standard deviation range of AN under respective predation conditions. The *P* value significance levels (two-sided one sample *t-*tests, *P* > .05 = ns, *P* < .05 = ^*^, *P* < .01 = ^**^, *P* < .001 = ^***^) in comparison to AN-no is shown by red asterisks/text and comparison to ancestor under respective predatory pressures are shown by black asterisks/text. Experiments were repeated independently at least twice, N1–8 (*n* = 10 replicates), L1–8 (*n* = 10 replicates), H1–8 (*n* = 13 replicates), AN-no (*n* = 72 replicates), AN-low (*n* = 10 replicates) and AN-high (*n* = 62 replicates).

For the last (15th) evolution cycle, we enumerated the number of prey CFU/ml at pre-predation, post-predation and recovery phases to calculate the conversion factor of OD_600nm_ to CFU counts (Supplementary Table S3). The latter forms the basis for estimating mutation supply in a population during experimental evolution, as described in detail in the Supplementary Materials and Methods. We also isolated single clones from these evolved prey populations after the 15th evolution cycle for subsequent analysis, as described in detail in the Supplementary Materials and Methods.

### Predation assay

To evaluate predation resistance and growth dynamics of different prey lineages, predation assays were performed. Different *E. coli* prey strains were assessed: *E. coli* K-12 MG1655 population lineages evolved under high (H1–H8), low (L1–L8) and no (N1–N8) predatory pressure at the end of 15th evolution cycle (after recovery phase) were compared with ancestor *E. coli* K-12 MG1655. First, we exposed all prey lineages to the predation pressure under which they had evolved (Fig. 1). Second, we exposed all evolved lineages to the high predatory pressure condition (Fig. 2) to test whether media adaptation in N1–N8 contributes to increased growth under predation and whether putative resistance evolved under low predation pressure in L1-L8 also confers protection under high predation pressure. Moreover, to investigate the temporal aspect of evolution for lineages H1–H8, we performed predation assays (under high predatory pressure) at the end of the second and fifth evolution cycle in addition to the 15th cycle (Supplementary Fig. S2). All population-level predation assays featured five replicates per evolved lineage and were repeated on two separate days. Finally, to assess the effects of specific prey genes on predation resistance, predation assays were conducted with markerless single gene deletion mutants (Δ*envZ,* Δ*ompF,* Δ*truA,* Δ*waaB,* and Δ*waaF*) based on *E. coli* BW25113 (reference strain from KEIO library [28]). These strains were exposed to high and no predatory pressure conditions.

**Figure 2:**
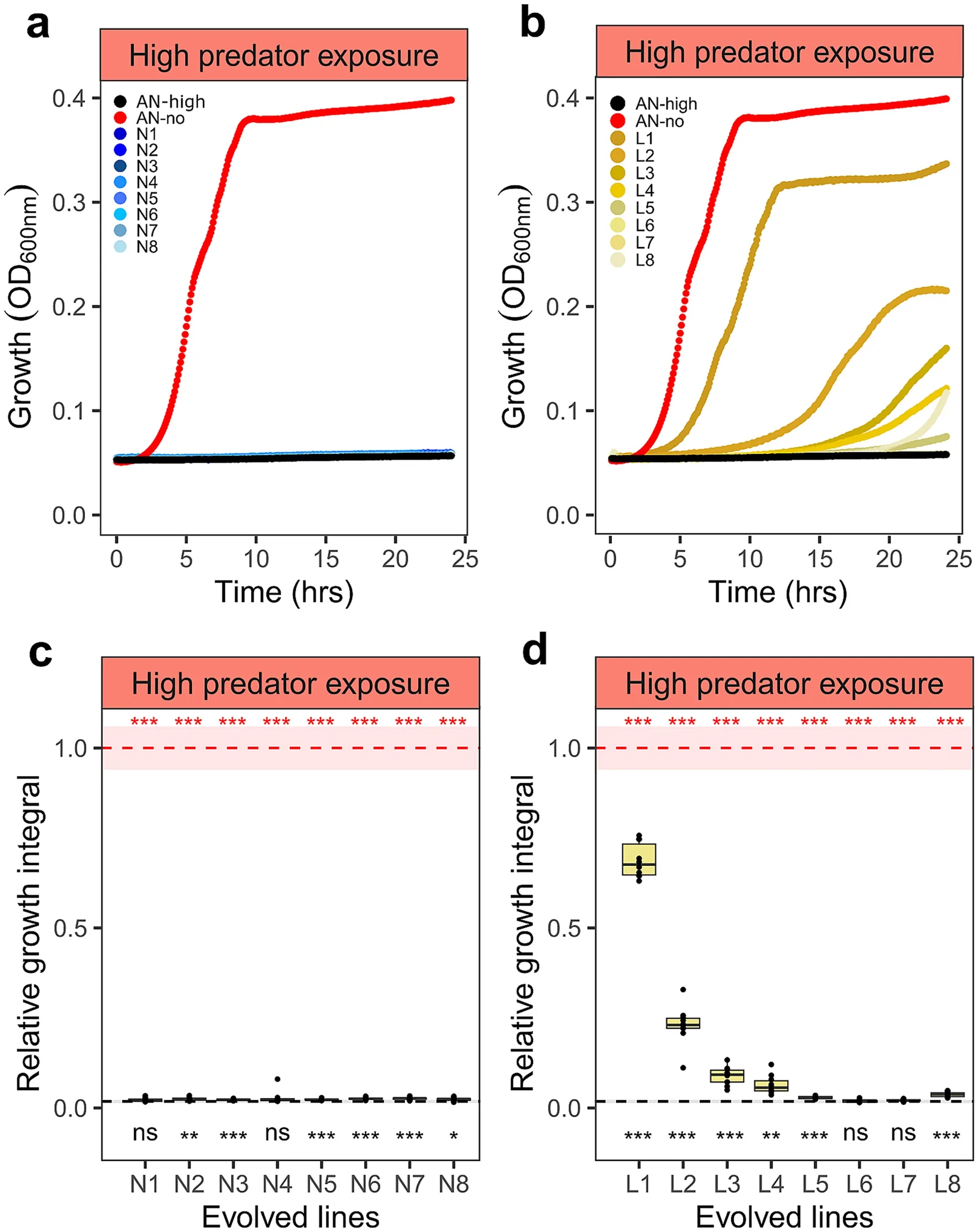
Predation resistance evolves only under predation. Growth dynamics of evolved prey from the 15th cycle: (a & b), growth curves measured as OD_600nm_ over time of eight independently evolved prey lineages under no (a, N1-8, blue) and low (b, L1-8, yellow) predatory pressure when exposed to high predatory pressure and compared with ancestor population under no (AN-no, red line) and high (AN-high, black line) predatory pressure. Each point of the growth curves represents average OD_600nm_ at that timepoint across replicates. (c & d) Relative growth integral of (a & b) defined as growth integral (area under the curve) divided by the growth integral of AN-no. Boxplots represent the median with 25th and 75th percentiles, and whiskers show the 1.5 interquartile range. Dotted lines and shaded areas indicate the mean and standard deviation range of the ancestor under no (AN-no, red) and high (AN-high, black) predatory pressure. The *P* value significance levels (two-sided one sample *t*-tests, *P* > .05 = ns, *P* < .05 = ^*^, *P* < .01 = ^**^, *P* < .001 = ^***^) in comparison to AN-no are shown by red asterisks/text, and to AN-high are shown by black asterisks/text. Experiments were repeated independently at least twice, N1–8 (*n* = 10 replicates), L1–8 (*n* = 10 replicates), AN-no (*n* = 72 replicates), and AN-high (*n* = 62 replicates).

Prior to the predation assay, respective evolved prey lineages or prey strains (evolved lineages, population- or single clone-based, single gene deletion, and KEIO reference strain) were grown from cryo stocks by inoculation in 50 ml YT broth and incubation at 37°C and 200 rpm for 16 h. Additionally, an overnight culture of ancestor prey was prepared from a single colony, at same growth conditions. The eight independently evolved prey lineages under each predatory pressure were assessed in parallel alongside ancestor prey. 10-ml prey overnight cultures were centrifuged at 5311× *g* for 3 min and subsequently washed with 10 ml of Ca-HEPES buffer. Then prey cells were resuspended in 6 ml of Ca-HEPES buffer and adjusted to OD_600nm_ = 0.03. A 24-h predator lysate was filtered through a 0.8-μm syringe filter (Whatman Puradisc FP 30, Cellulose Acetate, sterile, WHA10462240). For the predation assays, the same cultivation media was used as for the experimental evolution predation phase, comprising 15 ml of YT broth, 5 ml of 25 mM HEPES and 2 mM CaCl_2_ overall. We set up the predation assay in 96-well plates, in a total volume of 150 μl. Initially 119 μl of cultivation media was preset, and different volumes of the filtered predator lysate were added to the assay: 0 μl (no predator exposure), 4 μl (low predator exposure), or 16 μl (high predator exposure), depending on the evolved prey lineages and predatory pressure tested. Then we added 15 μl of the respective prey solution (OD_600nm_ = 0.03 in Ca-HEPES) and subsequently adjusted the final volume in reaction wells to 150 μl by adding Ca-HEPES (as proxy for predator lysate and prey resuspension), resulting in a final concentration of prey of OD_600nm_ = 0.003. We used the same ratio of prey and predator as in the predation phase of the prey evolution experiment. The plate was incubated in a plate reader (Tecan Infinite 200 Pro) at 29°C for 24 h. OD_600nm_ in the reaction wells was measured with 10 flashes every 5 min after shaking at 6-mm amplitude for 120 s.

We used OD_600nm_ as proxy for the biomass of prey cells, as the predator cells only contribute to a very minor fraction to OD_600nm_ based on the small size. The kinetic measurement of OD_600nm_ over 24 h, was analysed using an interactive platform for analysis of microbial growth data (https://statu.shinyapps.io/ExploreMicrobialGrowth/, version 2016.01.12) designed by Adin Ross-Gillespie at University of Zurich. We used Spline function to fit the kinetic growth data to extract the integral (area under the curve) and maximal growth increase rate. For the predation assays in cultivation media, we derived relative growth integral and growth rate of evolved prey lineages in comparison to the ancestor prey. For this we divided the growth integral or growth rate of evolved lineages under all predator conditions by that of ancestor when exposed to no predator, i.e. baseline. Values >1 or <1, indicate that evolved lineages perform better or worse than the baseline.

### Cell shape analysis by phase-contrast microscopy

*E. coli* K-12 lineages evolved under low, no, and high predatory pressure (after the 15th evolution cycle), *E. coli* K-12 MG1655 (ancestor), as well as *E. coli* K-12 BW25113, ∆*envZ* and ∆*truA* were regrown from cryo stocks in YT broth at 37°C and 200 rpm shaking overnight. From each culture 10 μl was applied onto a 1% agarose in Ca/HEPES buffer pad on a clean microscope slide for phase-contrast microscopy. Imaging was performed using a Leica DMi8 inverted microscope equipped with a 100× objective (HC PL APO 1.40 oil PH3), a Leica monochrome fluorescence DFC9000 gTC Camera System, and Leica Application Suite X Software Version 3.7.6.25997 (Leica microsystems). Exposure time was 100 ms with illumination intensity of 140. For each evolved lineage and ancestor strain *E. coli* K-12 MG1655 a minimum of 15 images were collected. For shape analysis of the evolved cells, software Fiji/ImageJ (Version 2.90/1.53t, Java 1.8.0322, 64-bit) [29, 30] was used with the plugin MicrobeJ (Version 5.13o (16)—beta) [31]. Area and length restrictions were set to 0.5–3 p^2^, width to 0.5–2 p^2^. After initial shape detection, masks were manually adjusted to observed cell size and a minimum of 300 cells per lineage/strain were analysed.

### Statistical analyses

We used general linear models for statistical analysis in R 4.2.3 [32, 33]. To assess whether relative growth integral and relative growth rate vary in response to predatory pressure we used analysis of variance (ANOVA) models. We further used two-sided one-sample *t*-tests to test for significant differences in relative growth integral or growth rate between evolved prey lineages and that of ancestor when exposed to either no, low or high predatory pressure. Reported *P*-values for *t-*tests were corrected for multiple testing using the Holm method for predation assays. We used a logistic regression model to test for a negative association (trade-off) between mean predation resistance under high predatory pressure (relative growth integral) and mean relative growth rate in the absence of predation. A detailed output of the statistical tests carried out in this article are available in the additional online material file “Online_statistics_table.xlsx”. Final compositions of figures were arranged in Inkscape 1.2.

### Genome sequencing and analysis of evolved prey population lineages

We sequenced the genomes of the 24 evolved prey lineages at the population level (N1–N8, L1–L8, H1–H8) and at the single clone level (48 cultures comprising six single clones per lineage H1–H8) after the 15th evolution cycle, alongside the ancestor *E. coli* K-12 MG1655 and compared them with reference *E. coli* K-12 MG1655 genome (refseq GCF_000005845.2). The full protocol of sample preparation from overnight cell growth, genomic DNA extraction, sequencing and base variant calling (evolved lineages versus ancestor) is described in the Supplementary Materials and Methods.

### Proteomic analysis of prey evolved under high predatory pressure

A shot-gun proteome was generated from the H1–H8 prey lineages evolved under high predatory pressure (collected after the 15th evolution cycle), the ancestor, and the ∆*ompF* and ∆*waaF* mutants in addition to reference strain BW25113. Three independent biological repeats of each *E. coli* lineage/strain were grown under conditions closely matching the predation phase of the evolution experiment, in the same medium but without addition of the predator. The full protocol of sample preparation, measurement and mass spectrometry data analysis are described in the Supplementary Materials and Methods. The frozen pellets of these cells were analysed by the Functional Genomic Center Zurich (FGCZ). Cell pellets were lysed, washed, and digested. The resulting peptides were washed, resolubilized, and analysed on an Orbitrap Fusion Lumos Mass spectrometer (Thermo Scientific) which was coupled to a M-Class UPLC (Waters) using the data-independent acquisition mode. The identification and quantification of proteins from MS data were performed using the DIA-NN workflow (version 1.8.2). A minimum of two unique peptides per protein had to be detected for inclusion in the differential expression comparison. The R package prolfqua [34] was used to analyze the differential expression and to determine group differences. P values were corrected for false discovery using the Benjamini-Hochberg method, for all quantifiable proteins. Heatmaps were generated in R with package Pheatmap [35], volcano plots were generated with Graph Pad Prism (version 6.07) and R 4.2.3 with package ggplot2 [32, 33, 36].

## Results

### Experimental evolution leads to increased prey growth

We experimentally evolved *E. coli* K-12 prey under no, low and high predatory pressures of *B. bacteriovorus* HD100 in eight-fold replication resulting in 24 distinct lineages. Experimental prey evolution was conducted for 15 cycles of predation and prey recovery (Fig. 1a, Supplementary Fig. S1). Thereafter, we subjected all evolved prey lineages to the predation pressure they experienced during experimental evolution. We found significant growth differences across the three predatory pressures (ANOVA: *F*_2,141_ = 8780.2, *P* < .0001). All *E. coli* lineages evolved without predator grew significantly better than the ancestor (red dotted line), which indicates media adaptation (Fig. 1b+e). For prey lineages evolved with predators, we observed that five low-predator evolved lineages (L1–L5, Fig. 1c+f) and all high-predator evolved lineages (H1–H8, Fig. 1d+g) showed significant growth recovery relative to the ancestor when subjected to predation (black dotted line). For most of these lineages (84.6%), growth recovery was partial, while only two lineages (H4, H5, 15.4%) showed complete recovery, compared to the unchallenged ancestor. Moreover, growth under predation was higher for prey lineages evolved under high than under low predatory pressure. Altogether, these results suggest that prey evolved partial resistance to predation and that higher predation pressure selected for higher levels of resistance.

To test if resistance arose early, we repeated the growth experiments shown in Fig. 1b–g with all high-predator lineages (H1–H8) from the second and fifth evolution cycle (Supplementary Figs S1 and S2). These analyses confirmed that predation-resistant phenotypes emerged early on during experimental evolution as all lineages could readily grow under high predation pressure at evolution cycle 2, with barely any growth differences existing compared to later evolution cycles 5 and 15 (Supplementary Figs S1 and S2). Hence, predation resistance evolved and spread early and once emerged within a prey lineage it persisted over generations.

### Predation and not media adaptation drives resistance evolution

In principle, media adaptation could be an explanation for the observed resistance phenotypes because faster growth would allow the prey to outpace the predator’s killing speed. If this hypothesis holds true we expect that no-predator evolved lineages, which showed highly improved growth, should be able to resist predation. We found no support for this hypothesis as all no-predator evolved lineages were still fully susceptible to predation (Fig. 2a and c). Instead, our results show that lineages L1–L5 (which evolved partial resistance under low predation) were also able to grow under high predatory pressure (Fig. 2b and d). We even observed slightly increased growth in a previously unnoticed lineage (L8). Together, these results indicate that adaptations are predation-specific and not driven by media adaptation.

### Trade-off between predation resistance and prey growth in the absence of predation

Antibiotic resistance is often associated with fitness costs in the absence of antibiotics [37, 38]. Here, we tested whether a similar trade-off occurs for *E. coli* in the context of predation resistance. To investigate this, we grew all evolved lineages without predators and calculated their growth rate relative to the ancestor. Relative growth rates differed significantly across predatory pressures (ANOVA: *F*_2,141_ = 460.42, *P* < .0001), and were highest in no-predator evolved *E. coli* lineages (Fig. 3a and d), followed by low-predator evolved lineages with growth rates similar to the ancestor (Fig. 3b and e) and high-predator evolved lineages with generally lower growth rate than the ancestor (Fig. 3c and f). When contrasting these growth rates against relative prey resistance (quantified as relative growth integral under high predatory pressure), we found a significant negative logistic correlation between predation resistance and prey fitness, indicating the predicted trade-off (Fig. 3g). Thus, our findings corroborate that *E. coli* resistance mechanisms are costly in the absence of *B. bacteriovorus* predation.

**Figure 3:**
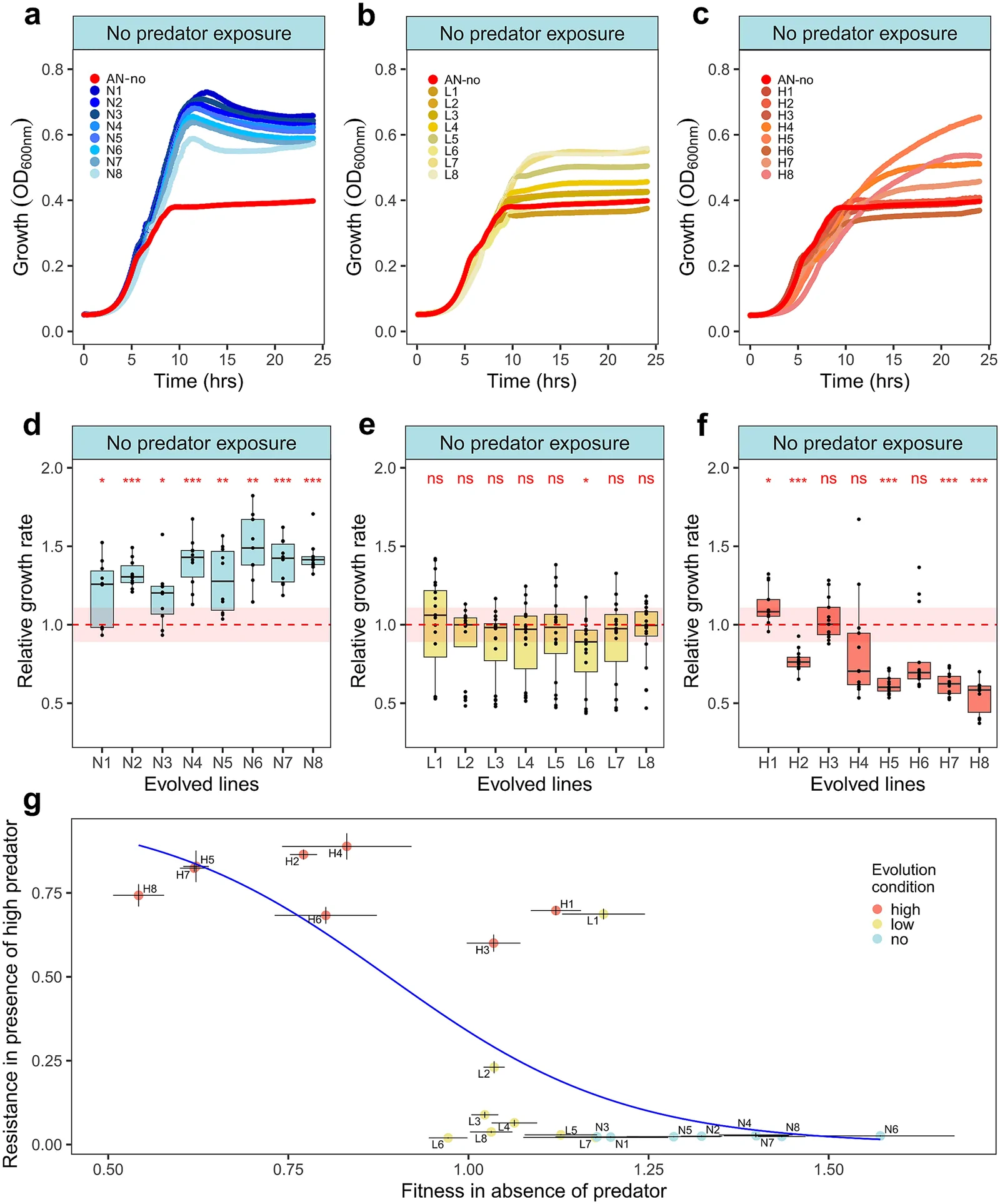
Prey resistance to bacterial predator is generally associated with negative fitness consequences. Growth dynamics of evolved prey from the 15th cycle: (a–c) growth curves measured as OD_600nm_ over time, of prey lineages evolved under no (a, blue), low (b, yellow) and high (c, red) predatory pressure, when exposed to no predatory pressure. Each point of the growth curve represents the average OD_600nm_ at that timepoint across replicates. Panel a is identical with Fig. 1a for a complete overview. (d–f) Relative growth rates of no (d), low (e), and high (f) predatory pressure evolved lines, when exposed to no predatory pressure. Relative growth rate is defined as maximum growth rate divided by the maximum growth rate of the ancestor under no predatory pressure (AN-no). Boxplots represent the median with 25th and 75th percentiles, and whiskers show the 1.5 interquartile range. Red dotted lines and shaded areas indicate the mean and standard deviation range of ancestor under no predatory pressure (AN-no). The *P* value significance levels (two-sided one sample *t*-tests, *P* > .05 = ns, *P* < .05 = ^*^, *P* < .01 = ^**^, *P* < .001 = ^***^) in comparison to AN-no are shown by red asterisks/text. Experiments were repeated independently at least twice, N1–8 (*n* = 10 replicates), L1–8 (*n* = 20 replicates), H1–8 (*n* = 13 replicates),AN-no (*n* = 72 replicates). Three outliers, one each from N6, L4 and L7 were excluded from panels d & e for better visualization but were included in the statistical analysis. (g) Trade-off between predation resistance and prey fitness. Fitness, quantified as mean relative growth rate under no predatory pressure (d–f) versus predator resistance, quantified as mean relative growth integral under high predatory pressure (Figs 1g and 2c–d). N1–8, L1–8, and H1–8 denotes prey lineages that evolved under no (blue), low (yellow) and high (red) predatory pressure respectively. A logistic regression function was fitted as a blue line to correlate the two variables (resistance versus fitness, Wald z-statistics = −2.336, *P* value = .0195). Black error bars represent the standard error of mean for the respective variables.

### High consistency in resistance levels among single clones

Based on the detected trade-off between predation resistance and population prey growth, we wondered whether there is heterogeneity in predation resistance among clones within prey populations. Consequently, we analysed the predation resistance profile of six randomly isolated clones from each of the eight high-predator lineages (15th cycle, 48 clones) and found that all clones showed consistently high predation resistance. This shows that clones did not separate into susceptible and resistant phenotypes within lineages (Supplementary Figs S3 and S4), but rather expressed uniform phenotypes. This result further indicates that the mechanisms conferring resistance to predation likely operate at the individual and not at the group level of the prey. Such group-level mechanisms were proposed to operate via the secretion of small soluble substances (quorum-sensing signals, indole, cyanide) [24–26, 39] and are expected to protect not only the evolved producers but also susceptible wildtype cells.

### Mutation frequency in the prey genome peaks under high predatory pressure

To uncover the genetic basis of putative resistance mechanisms, we sequenced all 24 evolved *E. coli* lineages (N1–8: no predation, L1–8: low predation, H1–8: high predation) after the 15th evolution cycle along with the ancestor. The Illumina sequencing reads were mapped to the reference genome of *E. coli* K-12 MG1655, and multi-sample variant calling was performed with a frequency cutoff of 5%. We focused on *E. coli* genes that had mutations in evolved lineages but not in the ancestor (Supplementary Fig. S5). We excluded mutated rRNA and transposase genes because they did not correlate with any of the three evolution conditions (Supplementary Fig. S6). Using these filters, we uncovered 43 mutated genes of which 17 occurred in N-lineages, 17 in L-lineages and 30 in H-lineages (Fig. 4a). A total of 26 mutated genes were exclusively found under predatory pressure (Fig. 4a), and thus reflect the top candidates potentially explaining resistance phenotypes. H-lineages had more unique gene mutations than L-lineages (18 vs. 5, Fig. 4b). Moreover, there were fewer high-frequency mutations (>70%) among L-lineages than among H-lineages (Fig. 4b), which is likely explained by the higher selection pressure. In addition, we estimated the mutation supply in the prey population based on the mutation rate, cell number, and number of cell divisions to assess the likelihood of mutations to arise in putative resistance genes. Using *ompF*, a frequently mutated gene, as an example, we found that mutation supply rates were high, such that it is likely for beneficial mutations to arise within a prey lineage in the first 24-h predation cycle (Supplementary Table S4).

**Figure 4:**
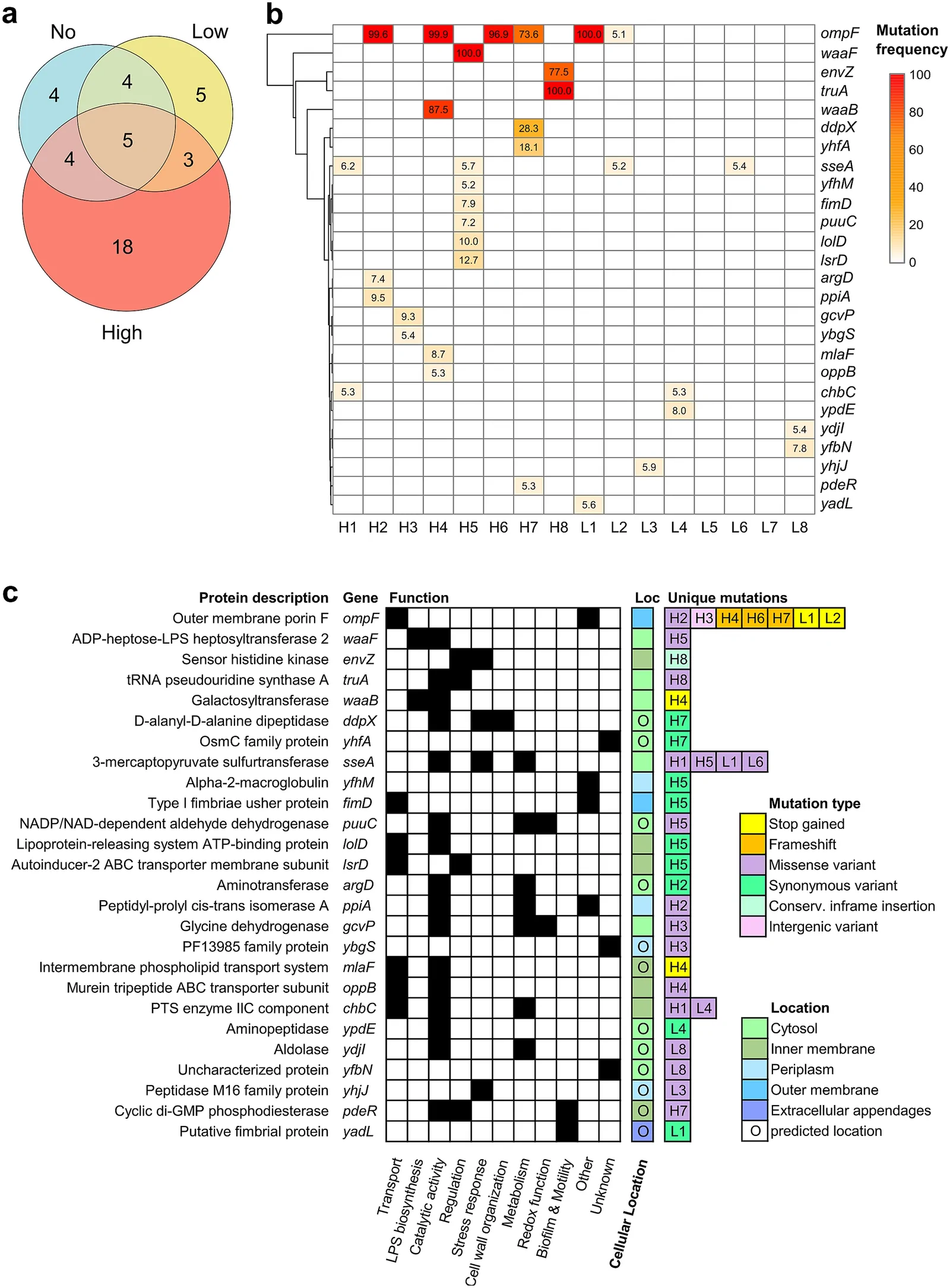
Gene mutational patterns in prey populations evolved under repeated predation pressure. Genes mutated in evolved *E. coli* K-12 lineages after the 15th evolution cycle in comparison to the ancestor. (a) Venn diagram showing the number of mutated *E. coli* genes that reached frequencies higher than 5% within a lineage, grouped according to no, low and high predatory pressure. Not included in this Venn diagram are mutated rRNA and transposase genes, which are listed separately (Supplementary Fig. S6). (b) Hierarchically clustered heatmap of mutation frequency differences relative to the ancestor of all the genes that exclusively mutated under high (H1–8) and/or low (L1–8) predatory pressure. (c) Overview of mutated genes and mutation types together with a description of the encoded proteins, their functions and cellular location (Loc) for all the genes that exclusively mutated under high and/or low predatory pressure, according to b.

### Outer membrane porin OmpF mutations arose repeatedly

Next, we conducted a cluster analysis (Fig. 4b) and analyzed the functions of the mutated gene products including mutation types and (predicted) amino acid changes (Fig. 4c, Supplementary Table S5). These analyses yielded *ompF*, encoding the outer membrane porin F, as the most frequently mutated gene. Four H-lineages (H2, H4, H6, and H7) and two L-lineages (L1, L2) showed mutations directly within the gene’s coding region. Moreover, lineage H3 had a point mutation in the upstream region of *ompF* (Supplementary Fig. S7) and lineage H8 had an inframe insertion in *envZ* (Supplementary Table S5), which encodes a sensor histidine kinase involved in OmpF regulation [40]. Mutation frequencies were high (73.6%–100%) in all lineages except in L2. The sequencing of six individual clones per H-lineage showed that mutations in *ompF* or *envZ* were present in all clones (Supplementary Fig. S8). Overall, there were no major differences in mutational patterns between clonal and population level sequencing (Supplementary Fig. S8), reinforcing the view of selective sweeps of a few beneficial mutations through populations. Mutation types in *ompF* include missense variants (H2), frameshifts (H4, H6, H7) and early stop codon gains (L1, L2) (Fig. 4b and c, Supplementary Fig. S7, Table S5), indicating that most observed mutations probably lead to OmpF function loss. AlphaFold predictions of the altered OmpF peptides and an overlay with the OmpF crystal structure supports the view that most mutations within *ompF* lead to a loss of function, apart from H2 (Supplementary Fig. S9).

### Proteomics reveals strong downregulation of OmpF and other structures on the prey surface

To test whether the genetic changes observed in evolved prey lineages under high predatory pressure affect protein levels, we conducted shot-gun proteomics with lineages H1–H8. Principal component analysis of the proteomics dataset showed three distinct clusters, whereby H1–H4, H6 and H7 formed one large cluster distinct from H5 and H8 (Fig. 5a). Differential analysis of protein expression levels was performed of all lineages vs. the ancestor (Fig. 5b, Supplementary Fig. S10). We put a special focus on the regulation of proteins encoded by the mutated genes (Fig. 5c), and the 20 most up- or downregulated proteins (Supplementary Fig. S11). The lineages H2–H4, H6, H7 all had mutations in or upstream of *ompF* (Fig. 4b and c) and we found that these lineages showed a strong downregulation of OmpF at the proteome level [log_2_(fold change) is: –8.2 (H2), −6.7 (H3), −7.5 (H4), −7.3 (H6), and −7.3 (H7)] (Fig. 5, Supplementary Fig. S10 and Supplementary Tables S6 and S7). H1 also showed a strong downregulation in OmpF protein levels (log_2_(fold change): −6.6, Fig. 5, Supplementary Fig. S10, Supplementary Tables S6 and S7) although no *ompF*-related mutations were detected.

**Figure 5:**
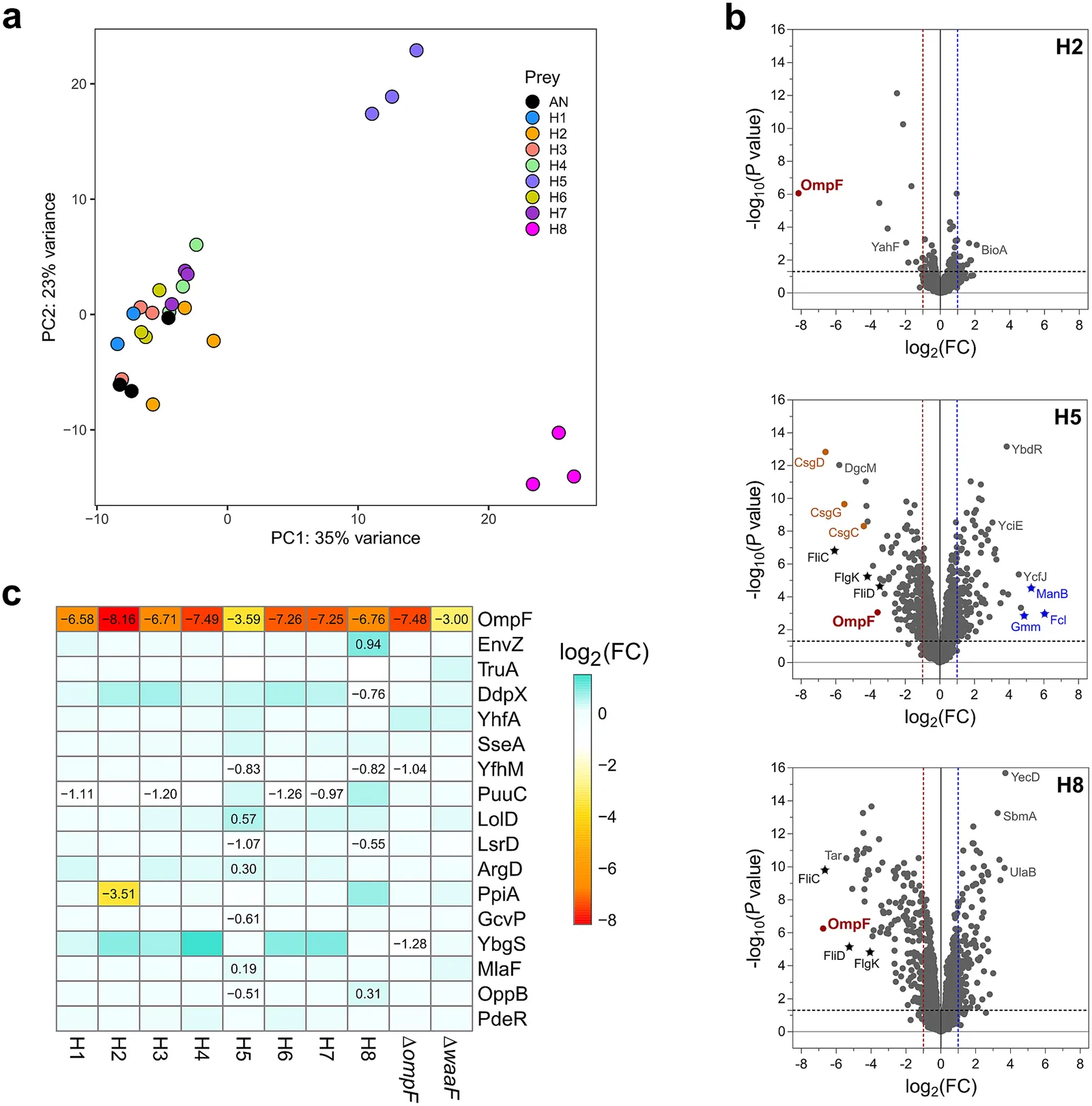
Proteomics of prey populations evolved under high predation pressure confirm strong downregulation of OmpF. Comparison of protein abundance in the eight prey lineages evolved under high predatory pressure (H1–H8) to the ancestor *E. coli* K-12 MG1655 and controls of *E. coli* Δ*ompF* and Δ*waaF* to the respective KEIO reference strain *E. coli* K-12 BW25113. (a) Principal component analysis of ancestor (AN) and eight prey lineages evolved under high predatory pressure (H1–H8) shows that the proteome of H5 and H8 are overall very different from the other lineages. (b) Selection of three volcano plots from proteomics analysis, showing H2 as a representative for evolved prey lineages with mutations in *ompF* leading to a downregulation on the proteome level. All volcano plots are shown in Supplementary Fig. S10. The dotted lines indicate log_2_(fold change, FC) of 1 (2-fold increase, blue) and −1 (two-fold decrease, red) in protein abundance. The black dotted line is the significance threshold set at -log_10_(adjusted *P* value) of 1.3, which equals an adjusted *P* value of .05. Highlighted are values for OmpF (red dots), curli assembly proteins (CsgC, D, G; orange dots) as well as flagella-associated proteins (FliC, FliD, FlgK; black stars) and sugar-converting proteins (Gmm, ManB, Fcl; blue stars). (c) Heatmap of log_2_(FC) for those proteins of which the genes were mutated exclusively under high predatory pressure and that were detected with at least two peptides in the proteome data set. Significant values of log_2_(FC) are shown numerically (adjusted *P* value below .05), non-significant values are only shown by color shading.

On the contrary, H5, which has a point mutation in *waaF* (mutation frequency: 100%), had a distinct proteome signature characterized by the down-regulation of surface structure proteins, like curli fibers (CsgC, CsgD, CsgG) and flagella-associated proteins (FliC, FliD, FlgK) (Fig. 5b and Supplementary Figs S10, S11 and Supplementary Table S6). Additionally, H5 also showed downregulation of OmpF although to a lower extent (log_2_(fold change) of −3.6, Fig. 5b, Supplementary Fig. S10, Supplementary Tables S6 and S7). This downregulation is likely a pleiotropic effect of the missense variant in *waaF*, since the proteome of Δ*waaF* (clean deletion) showed a similar downregulation of OmpF (log_2_(fold change): −3.0, Fig. 5c, Supplementary Figs S10 and S11, Supplementary Table S6). Finally, H8, which has mutations in *envZ* (mutation frequency: 77.5%) and *truA* (100%) had yet another distinct proteome signature characterized by downregulation of flagella-associated proteins FliC, FliD and FlgK and OmpF (log_2_(fold change): −7.48, Fig. 5c). In conclusion, our proteome analysis reveals major phenotypic changes involving drastic downregulation of OmpF in all lineages and other surface structures (curli fibers and flagella) in some lineages (H5 and H8). Some of these changes are caused by mutations in the respective genes, whereas other changes are the result of pleiotropic effects. Overall, the proteome data indicate that modification of surface structures is the key to predation resistance.

### OmpF absence confers resistance to predation

As OmpF is a known attachment site for phages and is known to be critical for effective *B. bacteriovorus* predation [15], we aimed to directly test whether a complete loss of OmpF confers predation resistance. Initially, we confirmed that *E. coli* KEIO reference strain (BW25113) was fully susceptible to *B. bacteriovorus* and grew well in the absence of predation (Fig. 6a and b), thus showing similar responses as the *E. coli* K-12 ancestor (Figs 1b, d and 2a, b). When subjecting the Δ*ompF* mutant to no and high predation pressures, we found this mutant to be completely resistant to predation, showing full growth recovery under high predator exposure (Fig. 6a and b). When comparing the proteome of Δ*ompF* to the reference, we observed (as expected), a drastic down-regulation of OmpF protein levels (log_2_(fold change): −7.48, Fig. 5c, Supplementary Table S6), comparable to many evolved prey lineages (H2, H4, H6, H7; Fig. 5, Supplementary Table S6). These results confirm that the downregulation of OmpF has a big impact on resistance.

**Figure 6:**
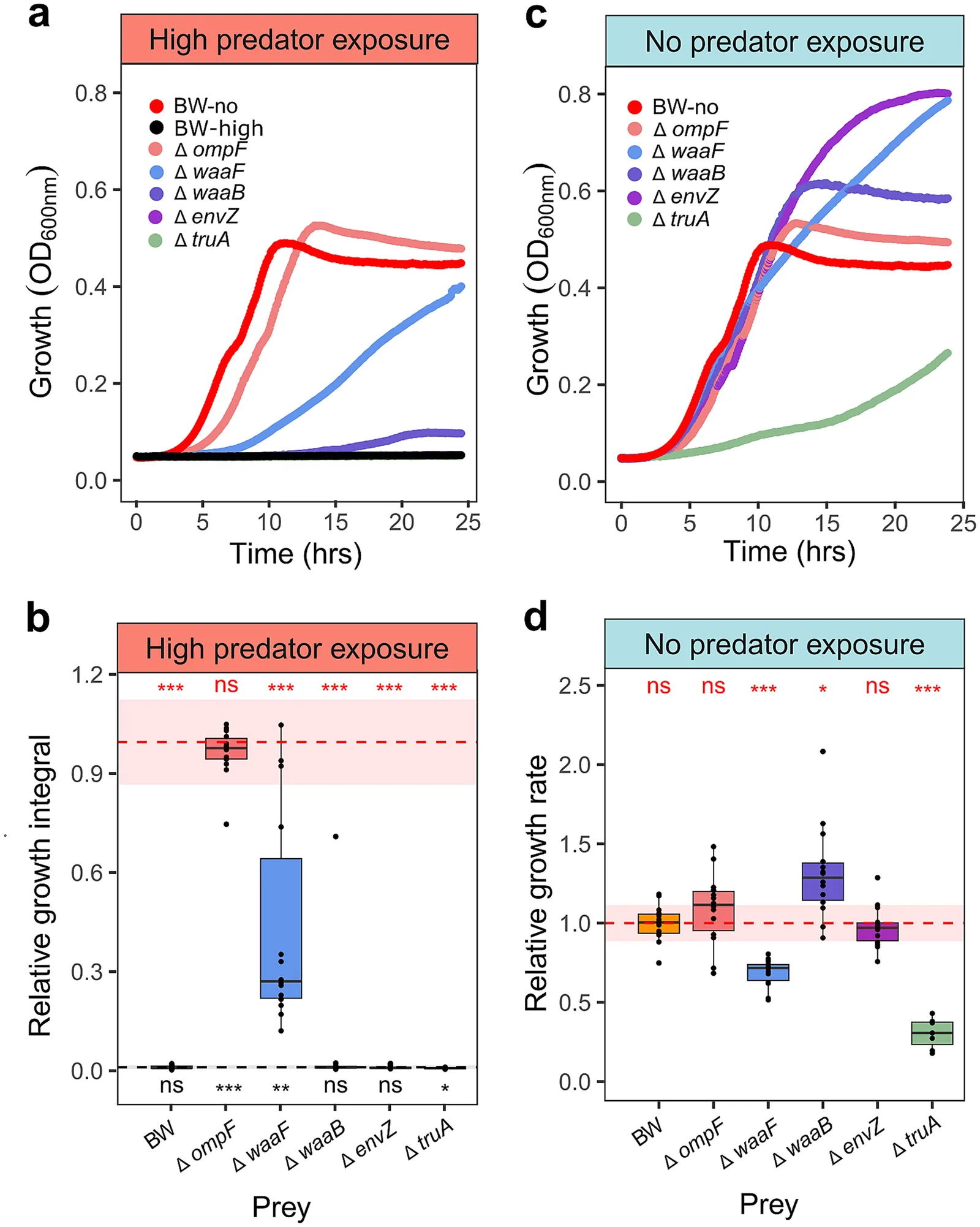
OmpF deletion in *E. coli* prey results in high predation resistance. Different *E. coli* clean-deletion mutant strains (Δ*ompF,* Δ*waaF,* Δ*waaB,* Δ*envZ,* Δ*truA*) were generated based on the KEIO library. *E. coli* BW25113 (BW) is the KEIO reference strain. (a + c) Growth curves measured as OD_600nm_ over time of the different *E. coli* deletion mutants exposed to either high predatory pressure (a) or no predators (c). Each point of the growth curve represents the average OD_600nm_ per timepoint across replicates. Experiments were independently repeated at least twice. Total replicate numbers for the various strains: Δ*ompF* (*n* = 14)*,* Δ*waaF* (*n* = 14)*,* Δ*waaB* (*n* = 14)*,* Δ*envZ* (*n* = 14)*,* Δ*truA* (*n* = 8), BW without predatory pressure (BW-no) (*n* = 14), and BW with high predatory pressure (BW-high) (*n* = 15). (b) Relative growth integrals from a. Relative growth integrals are calculated as growth integral (area under the curve) of the clean-deletion mutant with predatory pressure divided by the growth integral of BW-no. (d) Relative growth rates from c. Relative growth rates are calculated as the maximum growth rate of the clean-deletion mutant divided by the maximum growth rate of BW-no. Boxplots represent the median with 25th and 75th percentiles, and whiskers show the 1.5 interquartile range. Red and black dotted lines and shaded areas indicate the mean and standard deviation range of BW-no and BW-high respectively. The *P* value significance levels (two-sided one sample *t*-tests, *P* > .05 = ns, *P* < .05 = ^*^, *P* < .01 = ^**^, *P* < .001 = ^***^) in comparison to BW-no (red asterisks/text), and to BW-high (black asterisks/text).

### WaaF deletion confers partial resistance to predation

In lineage H5, a high frequency mutation occurred in *waaF* (100%, missense: Ala255Glu), encoding a heptosyltransferase involved in the biosynthesis of the LPS inner core [41]. Since our proteome analysis showed that mutations in this gene lead to the downregulation of several outer membrane structures, we aimed to directly test whether the loss of WaaF leads to predation resistance. We indeed found that a Δ*waaF* clean deletion mutant showed partial and significant resistance to predation (Fig. 6a and b). The same hypothesis could also hold true for the high frequency mutation observed in *waaB* (lineage H4, 87.5%, stop codon gained) encoding a galactosyltransferase involved in LPS biosynthesis. However, contrary to our expectation we did not find the Δ*waaB* clean deletion mutant to be resistant to predation (Fig. 6a and b). Instead, it showed significant growth improvements in the absence of predation (Fig. 6c and d). Because the mutation in *waaB* occurred together with an *ompF* mutation in lineage H4 we conclude that WaaB loss-of-function does not confer predation resistance but is beneficial in an already resistant lineage, showing the highest growth recovery under predation among all H-lineages, suggesting it to be a compensatory mutation (Fig. 1g).

### Mutations in truA cause costly prey morphology change

In addition to the phenotypic growth assays, genomics and proteomics analysis, we explored the cell morphology of the evolved lineages. Phase-contrast microscopy revealed that cells from lineage H8 no longer separated properly after cell division resulting in cells being thinner and longer (Supplementary Fig. S12a–c). No such abnormalities in cell morphology occurred in the other H- and L-lineages (Supplementary Figs S12 and S13). In lineage H8, in addition to the *envZ* mutation (77.5%) a high frequency mutation occurred in *truA* (100%, missense: Val200Ala). The *truA* gene encodes the tRNA pseudouridine synthase A, important for protein synthesis [42]. To assess the effects of these high frequency mutations on resistance and cell morphology, we generated deletion mutants. Deletion of *truA* did not result in predation resistance but was rather associated with severe fitness costs in the absence of predation (Fig. 6) and exhibited cell division abnormalities (Supplementary Fig. S12d), matching previously reported results on the effect of *truA* disruption [43]. We repeated the above experiments with a Δ*envZ* clean deletion mutant and found that this mutant was still susceptible to predation, in agreement with previously published data [15] (Fig. 6a and b). This is unsurprising because the experimentally evolved *envZ* mutant involved an inframe insertion [insertion of Lys–Thr–Trp–Leu between Leu135 and Lys136] and not a deletion. We hypothesize that the insertional mutation in our experiment result in lower levels of OmpF, which is confirmed by our proteomics data (log_2_(fold change) = −6.76, Fig. 5b and c, Supplementary Fig. S10 and Supplementary Table S6), and that functional changes in EnvZ, but not loss-of-function, could confer predation resistance. Further, our mutation frequency data suggest that the missense mutation in *truA* happened first and was associated with a drop in final OD_600nm_ after the recovery phase of early evolution cycles 1–5. Growth only recovered in later cycles, likely due to the additional mutation in *envZ*, probably partly compensating the fitness drop (Supplementary Fig. S1b). Altogether, our results indicate that resistance to predation may occur through cell morphology changes. However, such events might be rare due to the severe fitness costs typically associated with changes in fundamental cell physiological processes.

## Discussion

Antibiotic surveillance data tell us that bacterial pathogens have evolved resistance to every single drug used in clinics. Nowadays, it is therefore imperative to understand putative mechanisms and paths to resistance already during the development of new antibacterials [44, 45]. In our study, we performed this task for the predatory bacterium *B. bacteriovorus*, which is regarded as a potential living antibiotic preying on human pathogens. We found that the model prey *E. coli* K-12 MG1655 consistently evolved resistance against predation through two main mechanisms: mutations in the outer membrane porin OmpF (several independent events) that lead to its partial or complete down-regulation, and a mutation in WaaF (one event), an enzyme involved in outer membrane LPS biosynthesis that was associated with the pleiotropic down-regulation of curli fibers, flagellar structures, and the OmpF porin. These mechanisms suggest that selection acts on cell-membrane-associated traits that form the first line of contact between prey and predators. Although the precise mechanism of resistance still needs to be unraveled, the mutational targets revealed in our study match those observed to be under selection against bacteriophages [46, 47] (loss/alteration of host receptors [48, 49]) and membrane-targeting polymyxin antibiotics (LPS modifications) [50].

OmpF seems to be a major target of evolution in *E. coli* when exposed to antibacterial agents such as *B. bacteriovorus* (this study, [15]), toxins (colicin [51]), antibiotics [52–54] but also certain bacteriophages [48, 49]. OmpF enables *E. coli* to import and export small molecules via passive diffusion and can serve as bacteriophage docking site [49]. OmpF expression is high under neutral pH, whereas high osmolarity leads to its downregulation [55], therefore altering its susceptibility towards antibacterial agents. However, its mechanistic role in the interaction with *B. bacteriovorus* is not yet resolved. Here, we propose three scenarios of how loss or impairment of OmpF could foster predation resistance. Firstly, change of OmpF could prevent predators to use the porin as a docking site for attachment [15], similar to phage invasion [48, 49]. Secondly, OmpF might serve as an “overpressure valve” equilibrating the osmotic pressure building up when *B. bacteriovorus* enters the prey periplasm. Loss of OmpF or a reduction in its small molecule transport rate might compromise this function and presumably slow down predator entry. Thirdly, loss of OmpF could reduce the entry of potential, hypothetical small molecules secreted by the predator. Such molecules could manipulate the prey during attachment and recent evidence indicates that *B. bacteriovorus* can kill the prey even without subsequent entry [56]. Among these hypotheses, the most likely scenario is the involvement of OmpF in the attachment of the predator to the prey, since Mun *et al.* [15] showed a delayed predator attachment in an *ompF* deletion mutant, whereas the overexpression of OmpF in *E. coli* BW25113 resulted in an increased predation rate [15].

The observed mutational landscape indicates that multiple independent molecular routes can lead to OmpF downregulation. In four evolved *E. coli* H-lineages the downregulation of OmpF was very likely caused by direct mutations within *ompF* itself (H2, H4, H6, H7), whereas in two other lineages down-regulation of OmpF was likely driven by mutations in regulatory elements (H3 and H8). Conversely, the sequencing data did not yield any obvious genetic basis for OmpF downregulation in H1 pointing at yet unknown regulatory cascades potentially allowing bacteria to transiently regulate their phenotype [22]. For instance, genetic or phenotypic switches could form the regulatory basis as shown in other microbial predator–prey interactions [57]. Altogether, our results combined with previous reports indicate that OmpF is a mutational hotspot in the context of resistance evolution against antibiotics, bacteriophages, and predatory *B. bacteriovorus*.

Important to note is that the relevance of OmpF for successful *B. bacteriovorus* predation seems to depend on the prey species [15]. For example, deletion of OmpF in *Salmonella enterica* did not reduce prey killing [15]. Instead, it was reported that the LPS core has a major influence on attachment [58, 59]. Our evolution experiment detected one prey lineage that evolved predation resistance based on mutating *waaF*, encoding an LPS-modifying enzyme. The latter resulted in a pleiotropic effect, leading to down-regulation of curli fibers, flagellar proteins, and OmpF. Whereas we suspect that predation resistance is due to a combined effect including the downregulation of OmpF, the results also suggest that LPS structure take on an important role in the interactions with predators. Work with bacteriophages revealed two opposite roles for LPS (particularly the long O-antigens): depending on their specific structure they may act as primary receptors for attachment or block attachment to the outer membrane [48, 49]. Several lines of evidence suggest that the dual role also applies to interactions with *B. bacteriovorus* [20, 58, 60]. First, a phage-tail fiber protein homolog of *B. bacteriovorus* was indeed shown to bind to multiple bacterial surface glycans/LPS in multiple prey species (*E. coli* K-235, *Serratia marcescens*, and *Proteus mirabilis*) [20]. Second, attachment to prey is slowed down when predators are confronted with complex O-antigens as opposed to shorter LPS only featuring the core oligosaccharides in several species (*S. enterica, E. coli, Vibrio cholerae*) [58, 60]. Our results indicate that evolutionary change in LPS structure can modulate prey–predator interactions as observed for bacteriophage–bacteria interactions. While our results provide initial insights into the predator–prey surface interactions, the specific mechanisms by which alterations in prey outer membrane remodeling prevents predation remain unclear and will be the subject of future research with a particular focus on the three scenarios outlined above.

At first sight, our results on repeated resistance evolution seem to dampen the hope for predatory bacteria to become sustainable antibacterial treatments. Nevertheless, our experiments also revealed good news. Particularly, the strong trade-off between resistance to predation and growth in the absence of predation highlights that genetically manifested predation resistance is costly, compromising cellular functionality. This phenomenon could limit the spread of resistant prey as they are weak competitors and probably cleared away more efficiently by macrophages. There are concepts to leverage such competitive constraints to increase treatment sustainability [37, 61]. A key condition for leveraging on competitive constraints is that the pathogen is eliminated before compensatory mutations arise. We observed two putative compensatory mutations in our experiments (mutation in *waaB* in the background of an *ompF* mutant; mutation in *envZ* in the background of a *truA* mutant). Clearly, more research is required to understand the propensity of resistant prey mutants (with and without compensatory mutations) to spread. Although our evolution experiment using a laboratory *E. coli* strain provides first insights into predation resistance, additional work on clinical strains growing under relevant host conditions (e.g. human, animal, or plant) are required to substantiate our findings.

It will be particularly important to test whether similar evolutionary patterns (type of mutants, compensatory mutants, mutation frequency, and selection) are detected when treating pathogenic and clinical *E. coli* isolates. Additionally, future research will need to embrace the genetic diversity existing among *E. coli* strains to understand whether and what potential genetic variations already pre-exist in a prey population or multi-species infection that might compromise the efficacy of this “living antibiotic”.

To conclude, we like to emphasize three important points regarding prey resistance against bacterial predation by *B. bacteriovorus*. First, resistance mechanisms do not need to offer complete protection to escape predation. Instead, it suffices to alter the balance between prey growth and killing rate. In our experiment, we detected a decrease in killing rate (by mutations in *ompF* and *waaF*), but no increase in prey growth rate. Second, resistance mechanisms can be multifactorial and based on pleiotropic effects. Third, while we focused on predation resistance through mutation and selection processes as means of *de novo* resistance evolution, previous work has reported “plastic” resistance phenotypes, occurring after short timescale exposures, offering transiently protection against *B. bacteriovorus* [22]. Such plastic effects also surface in response to colistin exposure (i.e. change in membrane polarity) [50] and might well have occurred in our experiments (e.g. in resistant prey lineages exposed to low predatory pressure with few low frequency genetic mutations). A key task for future studies is to integrate all these aspects and to understand the dynamics of prey resistance and predation escape under situations that are closer to clinical settings—e.g. using clinical pathogen isolates, assessing treatment efficacy in spatially structured human cell cultures, and allowing predators to co-evolve with the prey. Only with such integrative knowledge, we can ensure the long-term sustainability of *B. bacteriovorus* as a living antibiotic, efficient in targeting multi-drug-resistant pathogens, in clinically relevant contexts.

## Supplementary Material

Supplementary_material_wrag235

## Data Availability

The raw data (OD_600nm_ measurements over time, raw data on width and length of *E. coli* cells, microscopy images and R code used for analysis) used to generate the findings of this project have been deposited in the figshare repository (https://doi.org/10.6084/m9.figshare.28015271). The sequencing data for this study has been deposited in the European Nucleotide Archive (ENA) at EMBL-EBI under accession number PRJEB96842 (https://www.ebi.ac.uk/ena/browser/view/PRJEB96842). The mass spectrometry proteomics data have been deposited to the ProteomeXchange Consortium via the PRIDE [62] partner repository with the dataset identifiers PXD080500 & PXD080501.

## Requests for materials

Requests for materials should be addressed to S.G.H. and S.M.

## Notes

Present address: Subham Mridha, Department of Microbiology, Perelman School of Medicine, University of Pennsylvania, Philadelphia, PA-19104, United States.

Present address: Ove Christopher Mattmann, Institute of Education, University of Zurich, Zurich CH-8032, Switzerland.

Present address: Simona G. Huwiler, Institute for Infectious Diseases, Faculty of Medicine, University of Bern, Bern CH-3001, Switzerland.

## References

[1] Murray CJ, Ikuta KS, Sharara F, et al. Global burden of bacterial antimicrobial resistance in 2019: a systematic analysis. *Lancet* 2022;399:629–55. 10.1016/S0140-6736(21)02724-0 PMC8841637 35065702

[2] Pirnay J-P, Djebara S, Steurs G, et al. Personalized bacteriophage therapy outcomes for 100 consecutive cases: a multicentre, multinational, retrospective observational study. *Nat Microbiol* 2024;9:1434–53. 10.1038/s41564-024-01705-x PMC11153159 38834776

[3] Sockett RE, Lambert C. *Bdellovibrio* as therapeutic agents: a predatory renaissance? *Nat Rev Microbiol* 2004;2:669–75. 10.1038/nrmicro959 15263901

[4] Konwar AN, Hazarika SN, Bharadwaj P, et al. Emerging non-traditional approaches to combat antibiotic resistance. *Curr Microbiol* 2022;79:330. 10.1007/s00284-022-03029-7 PMC9510247 36155858

[5] Koskella B, Hernandez CA, Wheatley RM. Understanding the impacts of bacteriophage viruses: from laboratory evolution to natural ecosystems. *Annu Rev Virol* 2022;9:57–78. 10.1146/annurev-virology-091919-075914 35584889

[6] Atterbury RJ, Tyson J. Predatory bacteria as living antibiotics - where are we now? *Microbiology (Reading)* 2021;167. 10.1099/mic.0.001025 33465024

[7] Lai TF, Ford RM, Huwiler SG. Advances in cellular and molecular predatory biology of *Bdellovibrio bacteriovorus* six decades after discovery. *Front Microbiol* 2023;14:1168709. 10.3389/fmicb.2023.1168709 PMC10225642 37256055

[8] Saralegui C, Herencias C, Halperin AV, et al. Strain-specific predation of *Bdellovibrio bacteriovorus* on *Pseudomonas aeruginosa* with a higher range for cystic fibrosis than for bacteremia isolates. *Sci Rep* 2022;12:10523. 10.1038/s41598-022-14378-5 PMC9217795 35732651

[9] Upatissa S, Mun W, Mitchell RJ. Pairing colicins B and E5 with *Bdellovibrio bacteriovorus* to eradicate carbapenem- and colistin-resistant strains of *Escherichia coli*. *Microbiol Spectrum* 2023;11:e00173–23. 10.1128/spectrum.00173-23 PMC10269710 37036359

[10] Sun Y, Ye J, Hou Y, et al. Predation efficacy of *Bdellovibrio bacteriovorus* on multidrug-resistant clinical pathogens and their corresponding biofilms. *Jpn J Infect Dis* 2017;70:485–9. 10.7883/yoken.JJID.2016.405 28367880

[11] Dashiff A, Junka RA, Libera M, et al. Predation of human pathogens by the predatory bacteria *Micavibrio aeruginosavorus* and *Bdellovibrio bacteriovorus*: predation by *M. Aeruginosavorus* and *B. Bacteriovorus*. *J Appl Microbiol* 2011;110:431–44. 10.1111/j.1365-2672.2010.04900.x 21114596

[12] Jurkevitch E, Minz D, Ramati B, et al. Prey range characterization, ribotyping, and diversity of soil and rhizosphere *Bdellovibrio* spp. isolated on phytopathogenic bacteria. *Appl Environ Microbiol* 2000;66:2365–71. 10.1128/AEM.66.6.2365-2371.2000 PMC110534 10831412

[13] Im H, Choi SY, Son S, et al. Combined application of bacterial predation and violacein to kill polymicrobial pathogenic communities. *Sci Rep* 2017;7:14415. 10.1038/s41598-017-14567-7 PMC5663959 29089523

[14] Koval SF, Hynes SH. Effect of paracrystalline protein surface layers on predation by *Bdellovibrio bacteriovorus*. *J Bacteriol* 1991;173:2244–9. 10.1128/jb.173.7.2244-2249.1991 PMC207774 2007549

[15] Mun W, Upatissa S, Lim S, et al. Outer membrane porin F in *E. coli* is critical for effective predation by *Bdellovibrio*. *Microbiol Spectrum* 2022;10:e03094–22. 10.1128/spectrum.03094-22 PMC9769668 36445149

[16] Georjon H, Bernheim A. The highly diverse antiphage defence systems of bacteria. *Nat Rev Microbiol* 2023;21:686–700. 10.1038/s41579-023-00934-x 37460672

[17] Varon M . Selection of predation-resistant bacteria in continuous culture. *Nature* 1979;277:386–8. 10.1038/277386a0

[18] Gallet R, Alizon S, Comte PA, et al. Predation and disturbance interact to shape prey species diversity. Am Nat 2007;170:143–54. 10.1086/518567 17853998

[19] Gallet R, Tully T, Evans MEK. Ecological conditions affect evolutionary trajectory in a predator-prey system. *Evolution* 2009;63:641–51. 10.1111/j.1558-5646.2008.00559.x 19154363

[20] Caulton SG, Lambert C, Tyson J, et al. *Bdellovibrio bacteriovorus* uses chimeric fibre proteins to recognize and invade a broad range of bacterial hosts. *Nat Microbiol* 2024;9:214–27. 10.1038/s41564-023-01552-2 PMC10769870 38177296

[21] Chanyi RM, Ward C, Pechey A, et al. To invade or not to invade: two approaches to a prokaryotic predatory life cycle. *Can J Microbiol* 2013;59:273–9. 10.1139/cjm-2013-0041 23586752

[22] Shemesh Y, Jurkevitch E. Plastic phenotypic resistance to predation by *Bdellovibrio* and like organisms in bacterial prey. *Environ Microbiol* 2004;6:12–8. 10.1046/j.1462-2920.2003.00530.x 14686937

[23] Aharon E, Mookherjee A, Pérez-Montaño F, et al. Secretion systems play a critical role in resistance to predation by *Bdellovibrio bacteriovorus*. *Res Microbiol* 2021;172:103878. 10.1016/j.resmic.2021.103878 34492337

[24] Hoshiko Y, Nishiyama Y, Moriya T, et al. Quinolone signals related to *pseudomonas* quinolone signal-quorum sensing inhibits the predatory activity of *Bdellovibrio bacteriovorus*. *Front Microbiol* 2021;12:722579. 10.3389/fmicb.2021.722579 PMC8461301 34566925

[25] Dwidar M, Nam D, Mitchell RJ. Indole negatively impacts predation by *Bdellovibrio bacteriovorus* and its release from the bdelloplast. *Environ Microbiol* 2015;17:1009–22. 10.1111/1462-2920.12463 24673893

[26] Mun W, Kwon H, Im H, et al. Cyanide production by *Chromobacterium piscinae* shields it from *Bdellovibrio bacteriovorus* HD100 predation. *MBio* 2017;8:e01370–17. 10.1128/mBio.01370-17 PMC5736907 29259082

[27] Lambert C, Sockett RE. Laboratory maintenance of *Bdellovibrio*. *Curr Protoc Microbiol* 2008;9:7B.2.1–7B.2.13. 10.1002/9780471729259.mc07b02s9 18770540

[28] Baba T, Ara T, Hasegawa M, et al. Construction of *Escherichia coli* K-12 in-frame, single-gene knockout mutants: the Keio collection. *Mol Syst Biol* 2006;2006:0008. 10.1038/msb4100050 PMC1681482 16738554

[29] Schindelin J, Arganda-Carreras I, Frise E, et al. Fiji: an open-source platform for biological-image analysis. *Nat Methods* 2012;9:676–82. 10.1038/nmeth.2019 PMC3855844 22743772

[30] Rueden CT, Schindelin J, Hiner MC, et al. ImageJ2: ImageJ for the next generation of scientific image data. *BMC Bioinformatics* 2017;18:529. 10.1186/s12859-017-1934-z PMC5708080 29187165

[31] Ducret A, Quardokus EM, Brun YV. MicrobeJ, a tool for high throughput bacterial cell detection and quantitative analysis. *Nat Microbiol* 2016;1:16077. 10.1038/nmicrobiol.2016.77 PMC5010025 27572972

[32] R Core Team . R: A Language and Environment for Statistical Computing. Vienna, Austria, 2021.

[33] Wickham H, Averick M, Bryan J, et al. Welcome to the Tidyverse. *JOSS* 2019;4:1686. 10.21105/joss.01686

[34] Wolski WE, Nanni P, Grossmann J, et al. *Prolfqua* : a comprehensive *R* -package for proteomics differential expression analysis. *J Proteome Res* 2023;22:1092–104. 10.1021/acs.jproteome.2c00441 PMC10088014 36939687

[35] Kolde R . Pheatmap: pretty heatmaps. R Package Version 1.0.10. 2019.

[36] Wickham H . ggplot2: Elegant Graphics for Data Analysis, 2016th edn. New York: Springer-Verlag, 2016.

[37] Andersson DI, Hughes D. Antibiotic resistance and its cost: is it possible to reverse resistance? *Nat Rev Microbiol* 2010;8:260–71. 10.1038/nrmicro2319 20208551

[38] Andersson DI . The biological cost of mutational antibiotic resistance: any practical conclusions? *Curr Opin Microbiol* 2006;9:461–5. 10.1016/j.mib.2006.07.002 16890008

[39] Dwidar M, Jang H, Sangwan N, et al. Diffusible Signaling factor, a quorum-sensing molecule, interferes with and is toxic towards *Bdellovibrio bacteriovorus* 109J. *Microb Ecol* 2021;81:347–56. 10.1007/s00248-020-01585-8 32892232

[40] Cai SJ, Inouye M. EnvZ-OmpR interaction and osmoregulation in *Escherichia coli*. *J Biol Chem* 2002;277:24155–61. 10.1074/jbc.M110715200 11973328

[41] Gronow S, Brabetz W, Brade H. Comparative functional characterization *in vitro* of heptosyltransferase I (WaaC) and II (WaaF) from *Escherichia coli*. *Eur J Biochem* 2000;267:6602–11. 10.1046/j.1432-1327.2000.01754.x 11054112

[42] Davis DR, Poulter CD. Proton-nitrogen-15 NMR studies of *Escherichia coli* tRNAPhe from HisT mutants: a structural role for pseudouridine. *Biochemistry* 1991;30:4223–31. 10.1021/bi00231a017 2021615

[43] Tsui HC, Arps PJ, Connolly DM, et al. Absence of hisT-mediated tRNA pseudouridylation results in a uracil requirement that interferes with *Escherichia coli* K-12 cell division. *J Bacteriol* 1991;173:7395–400. 10.1128/jb.173.22.7395-7400.1991 PMC209251 1938930

[44] Aminov RI . A brief history of the antibiotic era: lessons learned and challenges for the future. *Front Microbiol* 2010;1:134. 10.3389/fmicb.2010.00134 PMC3109405 21687759

[45] Davies J, Davies D. Origins and evolution of antibiotic resistance. *Microbiol Mol Biol Rev* 2010;74:417–33. 10.1128/MMBR.00016-10 PMC2937522 20805405

[46] Fang Q, Feng Y, McNally A, et al. Characterization of phage resistance and phages capable of intestinal decolonization of carbapenem-resistant *Klebsiella pneumoniae* in mice. *Commun Biol* 2022;5:48. 10.1038/s42003-022-03001-y PMC8758719 35027665

[47] Oechslin F . Resistance development to bacteriophages occurring during bacteriophage therapy. *Viruses* 2018;10:351. 10.3390/v10070351 PMC6070868 29966329

[48] Maffei E, Shaidullina A, Burkolter M, et al. Systematic exploration of *Escherichia coli* phage–host interactions with the BASEL phage collection. *PLoS Biol* 2021;19:e3001424. 10.1371/journal.pbio.3001424 PMC8594841 34784345

[49] Nobrega FL, Vlot M, De Jonge PA, et al. Targeting mechanisms of tailed bacteriophages. *Nat Rev Microbiol* 2018;16:760–73. 10.1038/s41579-018-0070-8 30104690

[50] Jochumsen N, Marvig RL, Damkiær S, et al. The evolution of antimicrobial peptide resistance in *Pseudomonas aeruginosa* is shaped by strong epistatic interactions. *Nat Commun* 2016;7:13002. 10.1038/ncomms13002 PMC5494192 27694971

[51] Jeanteur D, Schirmer T, Fourel D, et al. Structural and functional alterations of a colicin-resistant mutant of OmpF porin from *Escherichia coli*. *Proc Natl Acad Sci USA* 1994;91:10675–9. 10.1073/pnas.91.22.10675 PMC45084 7524100

[52] Thanassi DG, Suh GS, Nikaido H. Role of outer membrane barrier in efflux-mediated tetracycline resistance of *Escherichia coli*. *J Bacteriol* 1995;177:998–1007. 10.1128/jb.177.4.998-1007.1995 PMC176695 7860612

[53] Choi U, Lee C-R. Distinct roles of outer membrane porins in antibiotic resistance and membrane integrity in *Escherichia coli*. *Front Microbiol* 2019;10:953. 10.3389/fmicb.2019.00953 PMC6503746 31114568

[54] Delcour AH . Outer membrane permeability and antibiotic resistance. *Biochim Biophys Acta - Proteins Proteom* 2009;1794:808–16. 10.1016/j.bbapap.2008.11.005 PMC2696358 19100346

[55] Kenney LJ, Anand GS. EnvZ/OmpR two-component Signaling: an archetype system that can function noncanonically. *EcoSal Plus* 2020;9:10.1128/ecosalplus.ESP-0001-2019. 10.1128/ecosalplus.esp-0001-2019 PMC7192543 32003321

[56] Tyson J, Radford P, Lambert C, et al. Prey killing without invasion by *Bdellovibrio bacteriovorus* defective for a MIDAS-family adhesin. *Nat Commun* 2024;15:3078. 10.1038/s41467-024-47412-3 PMC11003981 38594280

[57] Van Gestel J, Koo BM, Stürmer VS, et al. *Bacillus subtilis* in defense mode: switch-like adaptations to protistan predation. *Proc Natl Acad Sci USA* 2025;122:e2518989122. 10.1073/pnas.2518989122 PMC12501158 40991432

[58] Varon M, Shilo M. Attachment of *Bdellovibrio bacteriovorus* to cell wall mutants of *salmonella* spp. and *Escherichia coli*. *J Bacteriol* 1969;97:977–9. 10.1128/jb.97.2.977-979.1969 PMC249794 4886305

[59] Schelling M, Conti S. Host receptor sites involved in the attachment of *Bdellovibrio bacteriovorus* and *Bdellovibrio stolpii*. *FEMS Microbiol Lett* 1986;36:319–23. 10.1111/j.1574-6968.1986.tb01718.x

[60] Duncan MC, Forbes JC, Nguyen Y, et al. *Vibrio cholerae* motility exerts drag force to impede attack by the bacterial predator *Bdellovibrio bacteriovorus*. *Nat Commun* 2018;9:4757. 10.1038/s41467-018-07245-3 PMC6232129 30420597

[61] Wale N, Sim DG, Jones MJ, et al. Resource limitation prevents the emergence of drug resistance by intensifying within-host competition. *Proc Natl Acad Sci USA* 2017;114:13774–9. 10.1073/pnas.1715874115 PMC5748215 29233945

[62] Perez-Riverol Y, Bandla C, Kundu DJ, et al. The PRIDE database at 20 years: 2025 update. *Nucleic Acids Res* 2025;53:D543–53. 10.1093/nar/gkae1011 PMC11701690 39494541

