# Supplementary material for: Outer membrane changes enable evolutionary escape from bacterial predation
Source: ISME J. 2026 Sep 16;20(1):wrag235. doi: 10.1093/ismejo/wrag235 (PMC13704779; doi:10.1093/ismejo/wrag235)
Supplement: Supplementary file 1 [file supplementary_material_wrag235.zip › EXPLORE_SI_v21_fin_noLineNr_LitNonlinked.docx]

**Supplementary data for**

**Outer membrane changes enable evolutionary escape from bacterial predation**

**Subham Mridha, Ove Christopher Mattmann^1^, Sarah-Luise Loose^1^, Saskia Isele, Bernd Roschitzki, Rolf Kümmerli and Simona G. Huwiler.**

^1^ O. Chr. M. and S.-L. L. contributed equally to this work.

Corresponding authors: Subham Mridha and Simona G. Huwiler

Email: subhammridha@yahoo.com & simona.huwiler@unibe.ch

**This PDF file includes:**

Supplementary Methods & Materials	page 2-9

Figures S1 to S14	page 10-25

Tables S1 to S5	page 26-28

Table S6	legend on page 29, xls-tables online in
 Supplementary materials

Table S7	page 30

SI References	page 31-32

**Other supporting materials for this manuscript include the following:**

Tables containing all statistical analysis results for the figures are in file “Online_statistics_table.xlsx”.

all raw data (OD_600nm_ measurements over time, raw data on width and length of *E. coli* cells, R code used for analysis) used to generate the findings of this project have been deposited in the figshare repository (doi: 10.6084/m9.figshare.28015271).

Supplementary Materials and Methods

**Revival, cultivation of *Bdellovibrio bacteriovorus* HD100**

We followed generally the protocol by Lambert & Sockett, 2008 [1]. To revive *B. bacteriovorus* HD100 from frozen stocks, we used YPSC overlay plates. YPSC bottom agar plates are composed of 0.25 g MgSO_4_ [Sigma], 0.5 g CH_3_COONa [Sigma], 1 g yeast extract [Bacto], 1 g peptone from meat, peptic digest [Sigma], 10 g agar [Sigma], per liter Mili-Q water, at pH 7.6, with added 0.25 g l^-1^ CaCl_2_.2H_2_O [Merck] after autoclaving. Then 5 ml of melted YPSC top agar (YPSC with 6 g l^-1^ agar) at 55°C was mixed with 150 μl late-log *E. coli* K-12 MG1655 in a sterile 7-ml Bijou tube (Thermo Fisher Scientific) and poured on top of the YPSC bottom agar plate. After solidifying, 3 drops of 30 μl thawed predator stock from -80°C were placed onto the YPSC top agar and incubated at 29°C for 5 days. From the lytic zones of these YPSC overlay plates (indicative of *B. bacteriovorus* growth), material was transferred into 2 ml of Ca-HEPES buffer (5.94 g HEPES free acid [Sigma], 0.294 g CaCl_2_.2H_2_O [Merck], per liter Mili-Q water, buffered at pH 7.6) and supplemented with 150 μl of late-log *E. coli* K-12 MG1655 at OD_600nm_ = 4.0 and incubated for 66 h at 29°C and 200 rpm. For the successive growth of predator, we transferred 50 μl of the previous lysate into 2 ml of Ca-HEPES buffer and 150 μl of overnight prey culture at OD_600nm_ = 4.0. To ensure all *E. coli* prey cells were lysed after 66- or 24-h incubation in the lysate and free-swimming *B. bacteriovorus* had a fast-swimming speed suitable for fast predation, an aliquot was checked with a phase-contrast widefield microscope at 100x magnification.

**Generation of markerless deletion strains of *E. coli* KEIO library**

Markerless single deletion mutants in *E. coli* (Δ*envZ,* Δ*ompF,* Δ*truA,* Δ*waaB,* and Δ*waaF*) were constructed of the respective strain from the KEIO collection (based on *E. coli* BW25113) [2]. To achieve this, the kanamycin resistance cassette (present in the KEIO collection to disrupt the individual genes) was removed to enable predation assays with kanamycin-sensitive *B. bacteriovorus* HD100. The kanamycin cassette was excised via pCP20 [3], expressing an FLP-FRT recombinase. To generate electrocompetent cells, and excise the kanamycin cassette, several protocols from the Barrick lab were adapted [4, 5]. To prepare electrocompetent cells the desired *E. coli* strain in YT broth with 30 μg/ml kanamycin was incubated for 16 h at 37°C and 200 rpm. The stationary phase culture was used to inoculate at least 10 ml of YT broth with 30 μg/ml kanamycin at optical density with absorbance at 600nm (OD_600nm_) of 0.05 and grown at 37°C and 200 rpm. At an OD_600nm_ of ~ 0.6 the culture was centrifuged for 5 minutes at 6000 x *g* and 4°C, and the supernatant was discarded. The resulting pellet was washed 4 times with 10% ice-cold glycerol of the same volume as the initial inoculation volume. Finally, the pellet was resuspended in 10% glycerol at 100x concentration, and 50 μl aliquots stored at -80°C. These electrocompetent *E. coli* cells were thawed on ice and a maximum of 100 ng of plasmid pCP20 was added to the cells. After gentle mixing, the sample was transferred into a cooled 0.2-cm cuvette (Bio-Rad), and electroporated at 2.5 kV with a MicroPulser electroporator (Bio-Rad). Then, the sample was resuspended in 500 μl warm SOC medium and incubated for 30 min at 30°C and 120 rpm. The cells were plated on YT agar plates with 100 μg/ml ampicillin and grown at 29°C for at least 16 h. To select for loss of heat-sensitive plasmid pCP20 after recombination, a single colony from a YT agar plate with 100 μg/ml ampicillin was picked to inoculate 5 ml of YT broth, and grown for 16 h at 43°C, shaking at 200 rpm. 50 μl of a 10^-6^ dilution of this stationary phase culture was plated on YT agar without antibiotics for single clones. To screen for successful recombination and loss of plasmid, six colonies were selected and streaked out on YT agar with 30 μg/ml kanamycin, YT agar with 100 μg/ml ampicillin, and YT agar without antibiotics in this order. The YT plates without and with kanamycin were incubated at 37°C, the YT plates with ampicillin at 29°C for at least 16 h. To screen for successful excision of the kanamycin cassette, recombinants with sensitivity to both antibiotics were grown in 5 ml of YT broth, incubated for 16 h at 200 rpm and 37°C. Of these cultures, 20% glycerol stocks were made and stored at -80°C. To control correct excision of the kanamycin cassette, PCR was used to assess correct product size (Primers in Supplementary Table S2). Additionally, correct scar regions were confirmed by sanger sequencing (Microsynth AG, CH).

**Experimental prey evolution**

***General setup***

In the experimental prey evolution, *E. coli* K-12 MG1655 was used as ancestral prey strain and subjected to three different predatory pressures (no, low, high) by *B. bacteriovorus* HD100 involving 15 alternate cycles of predation and recovery phase (Fig. 1a & Supplementary Fig. S14).

***I) Initial growth of E. coli K-12 MG1655 ancestor***

Prior to experimental evolution, we prepared overnight culture of *E. coli* K-12 MG1655 from a single colony. From this culture, we prepared 20% glycerol stocks and stored them in -80°C, to be used as reference for the ancestor strain (these ancestor glycerol stocks were used in the predation assays). To start the experimental prey evolution, we harvested 10 ml of this *E. coli* K-12 ancestor prey culture by centrifugation at 5311 x g for 3 minutes. The prey cells were resuspended in 0.8% NaCl and adjusted to OD_600nm_ = 0.03. The prey and predator cultures were mixed in appropriate amounts in the subsequent predation phase.

***II) Predation phase***

A 24-hr lysate of *B. bacteriovorus* HD100 (described above in predator cultivation) was filtered through a 0.8-μm syringe filter (Whatman Puradisc FP 30, Cellulose Acetate, sterile, WHA10462240) to remove prey debris and potential remaining prey cells. For the predation phase of the experimental evolution the cultivation media contained 30 ml YT broth, 10 ml 25 mM HEPES buffer and 2 mM CaCl_2_ (for the total volume of 40ml) enabling growth of *E. coli* prey as well as predation by *B. bacteriovorus*. Experimental evolution was performed in 24-well plates (Falcon, clear, sterile, non-tissue culture-treated, ref 351147). We used 3 different volumes of filtered predator lysate: 0 μl (no predator exposure), 40 μl (low predator exposure) and 160 μl (high predator exposure) and inoculated 150 μl of ancestor prey culture at OD600nm = 0.03, in 1.15 ml of cultivation media and adjusted the final volume to 1.5 ml per well, by either adding Ca-HEPES (as proxy for predator lysate) or 0.8% NaCl (as proxy for prey suspension). The final concentration of prey in reaction wells was OD_600nm_ = 0.003.

The multiplicity of infection (ratio of predator (PFU/ml) vs prey cells (CFU/ml)) was ~ 2.5 for low and ~10 for high predatory pressure condition. For each predatory pressure condition, we had 8 replicates in total, distributed on two different 24-well plates, resulting in 8 independently evolving prey lineages for each experimental condition (no, low and high predatory pressure). As controls we made 4 replicates of each condition without prey, the condition of no-prey and no-predatory pressure was considered blank. We measured the starting OD_600nm_ of the reaction wells at the beginning of the predation phase in a multimode plate reader (Tecan Infinite M200 Pro), by shaking for 30 secs at 6 mm amplitude before measuring OD_600nm_ with 10 flashes. In the predation phase the 24-well plates were incubated at 29°C and 200 rpm shaking (Infors Novotron AK85 shaking incubator) for 24 h. After the 24 h predation phase, we measured the end OD_600nm_ of the reaction wells in the plate reader (same setting as before) and blank corrected the values against control wells containing only media (control wells without prey and predator). For each predation phase a new 24-h lysate of *B. bacteriovorus* HD100 was used to avoid co-evolution, next to removing *B. bacteriovorus* at the end of the predation phase by filtration.

***III) Separation of surviving E. coli from B. bacteriovorus from predation phase by filtration***

At the end of each predation phase, we separated surviving prey from predator by filtration, using sterilized 0.45-μm 24-well filter plates (PALL, AcroPrep Supor, ref 97031) with a multi-well plate vacuum manifold (PALL, ref 5017). The filter plates were primed with 4 ml YT broth in each well for 15 minutes, then vacuum manifold set at pressure -10 in Hg for 1 minute was used to drain the YT broth. Based on the end OD_600nm_ of the well after predation phase we transferred either 300 μl (when OD_600nm_ > 0.30) or 1 ml (when OD_600nm_ < 0.30) of the lysate from the predation phase plates onto the 0.45-μm 24-well filter plates and adjusted the final volume up to 1 ml with YT broth. The filtration was done at a vacuum pressure of -10 in Hg for 2 min. The residue (containing prey cells) was washed twice with 4 ml YT broth and filtered for 5 minutes, each at the same (previously mentioned) vacuum pressure. To facilitate filtration and avoid clogging of filter wells by prey debris we mixed each filter well by pipetting during the process. After filtration we resuspended the residue on top of the filter containing surviving prey cells in 1.5 ml YT broth and transferred it into new 24-well plates. We also measured OD_600nm_ of the resuspended residue using the plate reader (same setting as before).

***IV) Recovery phase for E. coli prey***

For the recovery phase, we regrew the prey cells which survived the predation phase. We inoculated 15 μl of the resuspended residue into 24 well plates containing 1.5 ml of YT broth per well (~1:100 dilution). Again, we measured the starting OD_600nm_ of the reaction wells (as previously). The 24-well plates were incubated at 37°C and 200 rpm for 16 h allowing surviving prey to grow in absence of the predator. To ensure no predatory pressure during this recovery phase (from a potential small amount of transferred predators) only YT broth, not containing any additional CaCl_2_ was used. After the 16-h recovery phase, we measured the end OD_600nm_ of the reaction wells in plate reader (same setting as before) and were blank corrected against control wells containing YT broth only. Then we prepared separate glycerol stocks of recovered prey containing 150 μl of evolved prey culture and 50 μl of 80% glycerol and stored them in -80°C in 96-well plates (Falcon, clear, non-tissue culture-treated, ref 351147).

***V) Starting new predation phase***

The recovered (evolved) prey was used to start the next evolution cycle, starting again with a predation phase as described for the first cycle using three different predatory pressures from a new 24-h lysate culture. After the predation phase, the surviving prey cells were again separated by filtering from the predator and the recovery phase followed as described for the first cycle.

We repeated the evolution cycles of predation and recovery phase overall 15 times. The predator used in every predation cycle, grown in Ca/HEPES at 29°C and 200rpm for 24 h, was always fed with 150 μl of OD_600nm_ = 4.0 overnight *E. coli* K-12 MG1655 ancestor strain. The cryo stocks of evolved prey lineages made after every recovery phase was later used in predation assays and for phenotypic and genetic comparison with the prey ancestor.

**Correlation of OD_600nm_ and viability and estimation of mutation supply in *ompF* gene during experimental evolution**

We started our experimental evolution with a theoretical estimate of 1’209’375 prey cells in each well, determined by plating of colony forming units (CFU) (Source data). We enumerated the number of prey CFU/ml for the last (15^th^) evolution cycle, at pre-predation (recovery phase of 14^th^ cycle), post-predation and recovery phase for two no-, two low- and three high-predation exposed prey lineages. The mean OD_600nm_ and CFU/ml counts across predation exposure and stages were used to calculate the according conversion factor between optical density and viability/ colony forming units (Supplementary Table S3). These values were then used to extrapolate the OD_600nm_ (Sheet F, xls of Source data) to CFU/ml counts for other evolution cycles and lineages. The estimated total CFU counts were used to calculate the mutation supply in the evolved population lineages. We followed a method earlier used by Vollenweider et. al (2024) [6].

To calculate the expected mutation supply in *ompF* gene after the first 24 h predation phase, we used the number of CFU at the start (CFUstart = 1’209’375) and end of the predation (CFUend) cycle. These values were used to calculate the number of generations $n=\frac{\log\left( \mathrm{CFUstart} \right)- log(CFUend)}{log 2}$. We used a mutation rate of 6.24 x 10^-10^ per nucleotide per cell division [7] and *ompF* gene size of 1089 bp for *E. coli* K-12 MG1655 (U00096.3) [8, 9]. Then we calculated the expected mutation supply in a population = $CFUstart*1089*6.24 x {10-}^{10}*2^{n}$ (Supplementary Table S4).

**Isolation of prey single clones**

Overnight cultures were made of evolved lineages under high predatory pressure (15^th^ evolution cycle) stored at -80°C, by extracting cell material (containing evolved population) from cryo stocks and inoculating 50 ml YT medium in 250 ml Erlenmeyer flasks, incubated at 37°C, 200 rpm for 16 h. We harvested prey cells from 10ml of these overnight cultures by centrifugation at 5311 x *g* for 3 min and subsequently washed them in 10 ml of Ca-HEPES. Then prey cells were resuspended in 6 ml of Ca-HEPES and adjusted to OD_600nm_ = 3.0, then we diluted the cultures to 10^-6^ and plated 100 μl on YT agar plates and incubated them at 37°C for 24 h. We randomly selected 6 single colonies from each evolved lineage and overnight cultures were made with these single colonies in 10 ml YT medium in 50 ml Falcon tubes incubated at 37°C, 200 rpm for 16 h. From this culture we prepared 20% glycerol stocks and stored them at -80°C. Prior to experiments, we made overnight cultures of the single colonies from cryo stocks, alongside their respective evolved lineage population and ancestor in 50 ml YT medium in 250 ml Erlenmeyer flasks, incubated at 37°C, 200 rpm for 16 hours. These single clone cultures were used for predation assays and genome sequencing.

**Genome sequencing and analysis of evolved prey population lineages**

We sequenced the genomes of the 24 evolved prey lineages (i.e. population, N1-N8, L1-L8, H1-H8) collected after the 15^th^ evolution cycle, and compared them with the ancestor prey genome. Overnight cultures were grown in YT broth (200 rpm at 37°C) from cryo stocks of evolved prey lineages from the 15^th^ recovery phase at the end of experimental evolution and separately from a single colony of the ancestor prey. From these cultures we extracted genomic DNA using GenElute Bacterial Genomic DNA Kit (Sigma, NA2110). The concentration of genomic DNA varied between 83.77 to 205. 407 ng/μl, which was sufficient for standard library preparation. The samples were then transferred to the Functional Genomic Center Zurich (FGCZ, Switzerland) for sequencing and base variant calling of the sequencing data of evolved lineages against the ancestor. For library preparation the TruSeq DNA Nano Sample Prep Kit v2 (Illumina, Inc, California, USA) was used in the succeeding steps. DNA samples (100 ng) were sonicated with the Covaris using settings specific to the fragment size of 350 bp. The fragmented DNA samples were size- selected using AMpure beads, end-repaired and adenylated. TruSeq adapters containing Unique Dual Indices (UDI) for multiplexing were ligated to the size-selected DNA samples. Fragments containing TruSeq adapters on both ends were selectively enriched by PCR. The quality and quantity of the enriched libraries were validated using TapeStation (Agilent, Waldbronn, Germany). An average fragment size of approximately 500 bp was achieved. The libraries were normalized to 10 nM in Tris-Cl 10 mM, pH 8.5 with 0.1% Tween 20. For sequencing and cluster generation the Novaseq 6000 (Illumina, Inc, California, USA) was used according to standard protocol. Sequencing were paired end at 2 X150 bp. Technical quality of Illumina paired-end (PE) reads was evaluated using FastQC (v0.11.9) and FastQScreen (v0.14.1). Raw reads were pre-processed using fastp (v0.20.0) with the following parameter: hard trimming of 10 nucleotide (nt) and 1 nt at 5' and 3 ends, respectively; quality trimming of both ends using a sliding window of 4 nt and mean quality of 20; trimmed reads shorter than 18 nt, and trimmed reads with mean read quality lower than 20 were filtered out. Pre-processed PE reads were aligned to the reference genome (*E. coli* K-12 MG1655 refseq GCF_000005845.2) using bwa (v0.7.17) with the "mem" algorithm. PCR duplicates were marked using Picard (v2.22.8, MarkDuplicates). Mapping results were quality controlled using SAMStat (v1.5.1), Qualimap (v2.2.1), and Picard (v2.22.8, CollectGcBiasMetrics). Multi-sample variant calling was performed using freebayes (V1.3.2) with frequency cutoffs of 5% and 1%, respectively. Only unique reads in primary alignments with alignment quality 30 and above (error probability of the alignment is 1 in 1000), and bases with quality 20 and above, were counted for variant analysis. Sites with multiple alternative alleles were split into multiple rows using bcftools. Variant annotation was performed using snpEff (v4.3) against reference gene models (NCBI, reference genome: ASM584v2, refseq: GCF_000005845.2). Called variants were filtered against the ancestor strain using a customized Perl script. Markers were retained, where all counted reads contain only the reference alleles in the ancestor strain. Per sample variant frequencies were then calculated for these markers using the customized Perl script. The resulting excel file from population variant calling was used for further analysis and representation by hierarchically clustered heatmaps on absolute mutation frequencies and their differences compared to the ancestor strain. Heatmaps were generated in R with Pheatmap [10]. We focused on gene mutations with a frequency more than 5%. If a particular gene had multiple mutation effects, we selected the mutation with the highest frequency. Mutation counts were added to a gene only if the mutation was located within the coding region of a specific gene (direct effects). This excludes downstream effects on adjacent genes. Mutations lying in intergenic regions were ignored due to their ambiguous impact on surrounding genes. An exception was made for the mutation located in the intergenic region between *asnS* and *ompF*. Since it is located 143 bp upstream the *ompF* transcription start site various *ompF* regulators bind adjacent to this locus and is likely to influence *ompF* transcription. The mutations in *ompF* were analysed in detail and the visualisation of OmpF amino acid sequence with changes in the different prey lineages was constructed in Excel (Microsoft Corporation, 2018) and compared with the ancestor strain. Uniquely mutated genes (without transposases and rRNA genes) of all genomes of prey lineages at the respective predatory pressure were plotted using R package VennDiagram [11]. Final figure composition was made in Inkscape 1.2.

**Genome sequencing and variant calling for prey single clones**

48 cultures were prepared from six single prey clones each of all eight prey lineages evolved under high predatory pressure (H1-H8) at the end of the 15^th^ evolution cycle. Overnight cultures of these single clones from lineages evolved under high-predatory pressure were inoculated from cryo stocks into 10 ml YT medium in 50 ml Falcon tubes, and incubated at 37°C, 200 rpm for 16 h. We harvested prey cells equivalent of OD_600nm_ = 10.0 from these cultures by centrifugation at 5000 *g* for 5 min, the cells were then washed (5000 g for 5 minutes) in 1 ml Phosphate buffered saline (PBS, Gibco) and resuspended into 0.5 ml DNA/RNA shield buffer (Zymo Research). This mixture was sent to MicrobesNG (UK, https://microbesng.com, Birmingham, UK), for DNA extraction, sequencing and base variant calling against reference genome *E. coli* K-12 MG1655.

DNA was extracted by lysing 4-5 µl cell suspension with 120 μL of TE buffer containing lysozyme (MPBio, USA), metapolyzyme (Sigma Aldrich, USA) and RNase A (ITW Reagents, Spain), and incubated for 25 min at 37°C. Proteinase K (VWR Chemicals, Ohio, USA) (final concentration 0.1 mg/mL) and SDS (Sigma-Aldrich, Missouri, USA) (final concentration 0.5% v/v) are added and incubated for 5 min at 65°C. Genomic DNA was purified using an equal volume of SPRI beads and resuspended in EB buffer (10 mM Tris-HCl, pH 8.0). The DNA was quantified with the Quant-iT dsDNA HS (ThermoFisher Scientific) assay in an Eppendorf AF2200 plate reader (Eppendorf UK Ltd, United Kingdom) and diluted as appropriate. Genomic DNA libraries are prepared using the Nextera XT Library Prep Kit (Illumina, San Diego, USA) following the manufacturer’s protocol with the following modifications: input DNA is increased 2-fold, and PCR elongation time is increased to 45 sec. DNA quantification and library preparation are carried out on a Hamilton Microlab STAR automated liquid handling system (Hamilton Bonaduz AG, Switzerland). Libraries are sequenced on a NovaSeq 6000 (Illumina, San Diego, USA) using a 250 bp paired end protocol. Reads are adapter trimmed using Trimmomatic version 0.30 [12] with a sliding window quality cutoff of Q15. De novo assembly is performed on samples using SPAdes version 3.7 [13], and contigs are annotated using Prokka version 1.11 [14]. Reads were aligned to the reference using BWA mem v0.7.17 [15] and are then processed using SAMtools v1.9 [16]. Variants were called using VarScan v2.4.0 [17] with two thresholds, sensitive and specific, where the variant allele frequency is greater than 10% respectively. The effects of the variants are predicted and annotated using SnpEff v4.3 [18]. The base variant calling was performed using the criteria of a sequence coverage of at least 3x and a frequency of at least 10% to generate a table reporting on the differences to the reference genome (NCBI, refseq: GCF_000005845.2). The resulting Microsoft Excel table obtained from Microbes NG was used to combine it with the population sequencing data based on the nucleotide position. Gene annotations were obtained from both population and clone sequencing data, with frequency more than 5%. Final figure composition was made in Inkscape 1.2.

**Proteomic analysis of prey evolved under high predatory pressure**

To investigate adaptation on the proteome level, we generated a shot-gun proteome of the eight prey lineage populations evolved under high predatory pressure at the end of the 15^th^ evolution cycle (H1-H8) and compared them to the proteome of the ancestor strain. As further controls, markerless single gene deletion mutants ∆*ompF* and ∆*waaF* of the KEIO library [2] were compared to their reference strain *E. coli* BW25113. These strains were grown in 100 ml Erlenmeyer flasks from cryo stocks in a rich medium containing 15 ml YT broth, 5 ml Ca/HEPES buffer (25 mM HEPES, 2 mM CaCl_2_, pH 7.6) and 0.15 ml 200mM CaCl_2_ to match closely the growth conditions during the predation phase of the evolution experiment, but without predator addition. Three independent biological replicates of each strain were generated and evaluated. Cultures were grown for 12 hours at 200 rpm and 29°C. After the 12-hr cultivation, a 0.6-ml culture aliquot was centrifuged at 16'000 x *g* for 5 min at 4°C in 1.5-ml Eppendorf safe-lock tubes, and the pellet resuspended in 1.2 ml PBS buffer (1x, pH 7.4, Gibco™ 10010015). Based on the OD_600nm_ measurement 1 x 10^9^ cells were spun down at 16'000 x *g* for 5 min at 4°C, assuming OD_600nm_ of 1.0 = 8 x 10^8^ cells/ml. The pellet was frozen in liquid nitrogen and transiently stored at -80°C until transfer to FGCZ.

Cell pellets were lysed using a tissue homogenizer (TissueLyser II, QUIAGEN) and around 150 mg of glass beads (425-500 µm, Sigma) in 100 µl 2% sodium dodecyl sulfate (SDS), 2% sodium deoxycholate (SDC), 50 mM triethylammonium bicarbonate (TEAB) buffer pH 8.5. After 2 cycles of protein extraction (2 min each, 30 Hz) the supernatant was incubated for 10 min at 95°C in a thermomixer at 800 rpm. As a final extraction step, we used High Intensity Focused Ultrasound (HIFU, vial-tweeter Hielscher GmbH) for 1 minute at 100% amplitude and 60% cycle time. Subsequently, the supernatant was reduced and alkylated for 30 min at 30°C and 700 rpm by the addition of Tris(2-carboxyethyl)phosphine and 2-chloroacetamide to a final concentration of 5 mM and 15 mM, respectively. Next, the samples were diluted with pure ethanol to achieve a final concentration of 60% ethanol by volume, and the subsequent steps were performed on a KingFisher Flex System (Thermo Fisher Scientific). 50 µg of each samples were incubated for 30 min at room temperature with a 10-fold excess of pre-washed carboxylated magnetic beads (w/w), which had been previously washed to remove any impurities. Subsequently, the beads were washed three times for three minutes with 80% ethanol. The enzymatic digestion was initiated by adding trypsin in 50 mM TEAB (pH 8.5) to the beads, which were then incubated at 37°C overnight. The peptide-containing solution was collected, and the magnetic beads were washed once with pure water. The two elutions were combined and dried down. The dried samples were resolubilized in 20 µl of MS-solvent (3% acetonitrile, 0.1% formic acid) and 1 µl of indexed retention time peptides (iRT; Biognosys AG) were spiked in each sample for future retention time calibration.

Mass spectrometry analysis was performed on an Orbitrap Fusion Lumos (Thermo Scientific) equipped with a Digital PicoView source (New Objective) and coupled to a M-Class UPLC (Waters). Solvent composition at the two channels was 0.1% formic acid for channel A and 0.1% formic acid, 99.9% acetonitrile for channel B. For each sample 2 μl of peptides were loaded on a Symmetry C18 trap column (100 Å, 5 µm, 180 µm x 20 mm, Waters) followed by a nanoEase MZ C18 HSS T3 column (100 Å, 1.8 µm, 75 µm x 250 mm, Waters). The peptides were eluted at a flow rate of 300 nl/min by a gradient from 5 to 22% B in 80 min, 32% B in 10 min and 95% B in 1 min. Samples were acquired in a randomized order. The mass spectrometer was operated in data-independent acquisition mode (DIA), acquiring a full-scan MS (350−1500 m/z) at a resolution of 60’000 at 200 m/z. Followed by HCD (higher-energy collision dissociation) fragmentation on 70 windows with 8 m/z width between 400 and 960 m/z. The MS2 resolution was set to 30’000 and a normalized collision energy of 33% was used. The samples were acquired using internal lock mass calibration on m/z 371.1012 and 445.1200. The mass spectrometry proteomics data were handled using the local laboratory information management system (LIMS) [19].

The identification and quantification of proteins from MS data was performed using the DIA-NN workflow (version 1.8.2). The following parameters for the library-free search were used: precursor charge +2 and +3, precursor mass range 350 m/z to 1500 m/z, fragment mass range 200 m/z to 1800 m/z, mass accuracy MS1 15 ppm, MS2 20 ppm, enzyme specificity trypsin/P allowing one missed cleavage, fixed modification carbamidomethylation of cysteine. The in-silico spectra were generated using a canonical protein database for *E. coli* and human, concatenated with common protein contaminants (o34855_96061spg_n_83333EcoliK12_20240613.fasta, ID9606 [human] and ID83333[*E.coli*], shared on PRIDE) and the maximum false discovery rate (FDR) was set to 0.1. To identify the peptides in OmpF (as shown in Supplementary Table S7), the DIA-NN workflow was performed without the function “match between runs”. For the quantitative analysis, the data was processed with DIA-NN workflow with the function “match between runs”, which is the standard in the field.

The R package prolfqua [20] was used to analyze the differential expression and to determine group differences, confidence intervals, and false discovery rates for all quantifiable proteins. We started with the report.tsv file, which was generated by DIA-NN using the quantitative DIA-NN workflow, and which reports the precursor ion abundances for each raw file. First, we determined protein abundances by aggregating precursor abundances to peptidoform abundances. Then, we employed the Tukeys-Median Polish to estimate protein abundances. Furthermore, before fitting the linear models, we transformed the protein abundances using the variance stabilizing normalization [21]. The *P* values were adjusted for multiple testing using Benjamini-Hochberg method. The resulting lists of differential expression comparison (H1-H8 versus ancestor and separately ∆*ompF* or ∆*waaF* versus BW25113) was filtered with Microsoft Excel to only contain proteins from *Escherichia coli* and a minimum of two unique peptides. Based on this data a manually assorted list was compiled in Microsoft Excel containing all proteins of genes as listed in Fig. 4b of which at least two peptides were identified and which genes were mutated in H1-H8 compared to the ancestor to generate Fig. 5c. Further, we identified top 20 upregulated and downregulated proteins other than ones reported in Fig. 5c, by applying a filter of 2 as minimal number of peptides, and fold change of bigger than 1 or lesser than -1, and adjusted *P* values of less than 0.05 to be included in the dataset. Heatmaps were generated in R with Pheatmap [10]. Data to generate the volcano plots were exported from FGCZ Shiny App Explore DE [22] with 2 as minimal number of peptides and false-discovery rate (FDR) adjusted *P* values to be included in the dataset. This data was filtered with Microsoft Excel to only contain proteins from *Escherichia coli,* and the gene name extracted from the description column. The volcano plots were generated based on this list in Graph Pad Prism (version 6.07) and R 4.2.3 and package ggplot2 [23–25]. For Supplementary Table S6, only significantly regulated proteins were retained with adjusted *P* values below 0.05 and a log_2_(fold change) bigger than 1 or smaller than -1. The principle component analyses (PCA) were exported from FGCZ Shiny App Explore DE [22] based on transformed data. Final adjustments on the labels on the PCA and volcano plot were made with Inkscape 1.2.

**AlphaFold prediction of partial OmpF structure**

ColabFold V1.5.5 [26, 27] was used for protein structure monomer and multimer prediction using Alphafold2 and AlphaFold2-multimer using MMseqs2 and HHsearch at default settings. The PDB files of the resulting predictions were overlayed with the OmpF crystal structure PDB number 2ZFG [28] as reference in PyMOL (version 3.1, Schrödinger) using built-in ‘align’ function. The ColorByRMSD script by Shandilya (<https://pymolwiki.org/index.php/ColorByRMSD>) was used to compute distances between aligned C-alpha atom pairs (B-factors of these residues) and to color them by a color spectrum, with blue specifying the minimum pairwise RMSD and red indicating the maximum. Unaligned residues are colored gray.

**Fig. S1.** **Growth of independently evolving prey lineages during experimental evolution.** A prey evolution experiment was conducted over 15 cycles, composed of an alternating predation and recovery phase, transferring only the evolved prey to the next evolution cycle (see Fig. **1a** for a detailed scheme). Evolved prey were then exposed to fresh predator in the subsequent predation phase. After separation of prey from predator, growth of prey in the absence of predator is defined as recovery phase. Growth was measured by OD_600nm_ of eight independently evolved *E. coli* K-12 prey lineages (1-8, different shapes) when subjected to no (N, blue), low (L, orange) and high (H, red) predatory pressure. **a,** Growth trajectories over 15 evolution cycles measured at the end of each 24-h predation phase of *E. coli* K-12 prey lineages under different predatory pressure. **b,** Growth trajectories over the 15 evolution cycles measured at the end of each 16-h recovery phase.

**Fig. S2.** **Phenotypic predation resistance occurs early and remains consistent over the course of experimental prey evolution.** Growth of prey lineages evolved under high predatory pressure (H1-H8) was measured at the end of 2^nd^, 5^th^ and 15^th^ evolution cycle to determine temporal progression. As a reference the ancestor was exposed to no (AN-no, intense red) and high predatory pressure (AN-high, black). **a-c** & **g-i,** Growth kinetics measured as OD_600nm_ when exposed to high **(a-c)** and no **(g-i)** predatory pressure. Each point of the growth curve represents the average OD_600nm_ at that timepoint across replicates. **d-f** & **j-l,** Relative growth integral defined as growth integral (area under the curve) divided by the growth integral of ancestor under no predatory pressure. Red dotted line and shadow indicate the mean and standard deviation range of ancestor under no predation (AN-no). Boxplots represent the median with 25^th^ and 75^th^ percentiles, and whiskers show the 1.5 interquartile range. The *P* value significance levels (two-sided one sample *t*-tests, *P*>0.05 = ns, *P*<0.05 = *, *P*<0.01 = **, *P*<0.001= ***) in comparison to AN-no is shown by red asterisks/text. Experiments were repeated independently at least twice, H1-8 2^nd^ cycle (n= 10 replicates), H1-8 5^th^ cycle (n= 10 replicates), H1-8 15^th^ cycle (n= 13 replicates), AN-no (n= 72 replicates) and AN-high (n= 62 replicates). Plot **c** & **f** in this figure are identical to Fig.1 **d** & **g**, plot **i** is identical to Fig. **3c**. Precise *P* values can be found in the extended online material (statistics table).

**Fig. S3.** **Growth kinetics of single clones in comparison to populations that evolved under high predatory pressure and the ancestor.** Growth kinetics measured as OD_600nm_ of single clones of high predatory pressure evolved lineages when exposed to **a-d, i-l**, high predatory pressure: H1 (**a**), H2 (**b**), H3 (**c**), H4 (**d**), H5 (**i**), H6 (**j**), H7 (**k**) and H8 (**l**), and to **e-h, m-p**, no predatory pressure: H1 (**e**), H2 (**f**), H3 (**g**), H4 (**h**), H5 (**m**), H6 (**n**), H7 (**o**) and H8 (**p**), compared with ancestor (AN-no, red and AN-high, black). Each point of the growth curve represents the average OD_600nm_ at that timepoint across replicates. Labels 1-6 denote single clones, H1-H8 denote populations that evolved under high predation pressure. H1-8 single clones (n= 3), H1-8 populations (n= 13), AN-no (n= 72) and AN-high (n= 62).

**Fig. S4.** **Variation in resistance between evolved prey single clones and populations. a**, Relative growth integral, defined as growth integral (area under the curve) of the single clones or populations divided by the growth integral of ancestor under no predatory pressure and **b**, relative growth rate, defined as maximum growth rate of the single clones or populations divided by the maximum growth rate of the ancestor under no predatory pressure of single clones (Nr. 1-6 in different colours) of H1-H8 from 15^th^ evolution cycle, under high and no predatory pressure respectively. Coloured circles represent the mean and coloured error bars represent the standard deviation for each clone. Boxplots represent the median with 25^th^ and 75^th^ percentiles for the evolved population, and whiskers show the 1.5 interquartile range. Black dots represent the outliers of H1-8 evolved population. Red dotted line and shadow indicate the mean and standard deviation range of ancestor under no predatory pressure (AN-no). H1-8 single clones (n= 3 replicates), H1-8 population (n= 13 replicates), AN-no (n= 72 replicates).

**Fig. S5. Diverse genome-wide mutation patterns within genes of all evolved *E. coli* K-12** **prey population lineages.** Hierarchically clustered heat map off all gene mutations with at least 5% frequency compared to *E. coli* K-12 MG1655 within a prey population lineage (excluding rRNA and transposase genes, which are shown in Supplementary Fig. S6) that evolved under high (H1-8), low (L1-8), and no (N1-8) predatory pressure at the 15^th^ evolution cycle, as well as the ancestor (AN). Population fitness (blue heatmap, average relative growth rate of the lineages under no predatory pressure) and resistance to predator (red heatmap, average relative growth integral of the lineages under high predatory pressure) is indicated on top (see Fig. 3).

**Fig. S6. Mutation frequency of the transposases and rRNA genes.** Heatmap with **a,** transposase and **b,** rRNA genes with at least 5% mutation frequency within a prey population that evolved under high (H1-8), low (L1-8), and no (N1-8) predatory pressure, at the 15^th^ evolution cycle as well as ancestor (AN), mapped/compared against *E. coli* K-12 MG1655.

**Fig. S7.** **Critical mutations occurring within the *ompF* gene resulting in the premature stop of OmpF in multiple evolved lineages.** Comparison of *ompF* gene of the *E. coli* K-12 MG1655 reference genome to the ancestor (AN), and different *E. coli* prey lineages evolved under high (H1-H8) and low predatory pressure (L1, L2). In lineage H3 no mutation within the coding sequence of *ompF*, but a point mutation in the intergenic region *ompF*-*asnS* upstream of *ompF* was detected (original C mutated to T at position 987125 at 100% of the population). Within lineage H7 two different types of mutations occurred, at different % within the population. Green bars represent identical translated amino acid (AA) sequences, grey bar represent different mutations at a specific locus, in many cases leading to a premature termination of gene translation (details in Supplementary Table S5).

**Fig. S8. Comparison of gene mutations between single clones and populations that evolved under high predatory pressure.** Hierarchically clustered heatmap of gene mutations with at least 5% frequency within any high predatory pressure evolved lineage populations H1-H8 (excluding rRNA and transposase genes, in Supplementary Fig. S6) at the 15^th^ evolution cycle in comparison to the ancestor (AN). The 21 genes shown are exclusively mutated under high predatory pressure in H1-H8 populations. Next to each column of population sequencing data of H1-H8, is variant calling data shown from single clones 1-6 originating from that population (e.g. H1.1-H1.6). The population and single clone mutations are matched based on nucleotide position. For populations, mutation frequencies denote the actual percentage of a particular variant within a population. For single clones’, the mutation frequencies shown are the highest mutation frequency of a particular variant, 0 indicates that variant is absent. The single clone sequencing data is analyzed with at least 3x coverage and a mutation frequency of at least 10%. All sequencing data was mapped against *E. coli* K-12 MG1655 prior to further analysis.

**Fig. S9.** **AlphaFold prediction of remaining OmpF structure in the *E.coli* lineages evolved under high predatory pressure H2, H4, H6, and H7.** *In silico* modelling is based on the mutations within *ompF* gene (Supplementary Fig. S7 & Table S5, mutation types are indicated below the lineages), using OmpF crystal structure PDB number 2ZFG [28] as scaffold. The wild type OmpF crystal structure (2ZFG) [28] is shown in grey in an overlay with the AlphaFold predictions. The colors indicate the RMSD (Root Mean Square Deviation) values between aligned Cα atom pairs, with blue representing regions of high structural similarity and red indicating regions of strong deviation. **a**, OmpF monomer from two different side views rotated by 180° around the barrel axis. The N-terminal helix (facing down) is the secretion signal which is cleaved off in the final membrane-integrated form. **b**, Top view of trimeric OmpF structure [28]. The asterisk at H6 indicates, that there was a problem with the alignment of the domain sequences because the mutant sequence is very short. Abbreviation: fs: frame shift.

**Fig. S10.** **Volcano plots of *E. coli* proteins in different lineages and strains confirm strong downregulation of OmpF.** Comparison of protein abundance in the eight prey lineages evolved under high predatory pressure (H1-H8) to the ancestor (AN) *E. coli* K-12 MG1655 and comparison of controls of *E. coli* Δ*ompF* and Δ*waaF* to the KEIO reference strain *E. coli* K-12 BW25113. Dotted lines show log_2_(Fold Change) of 1 (2-fold increase, blue) and -1 (2-fold decrease, red) in protein abundance. The black dotted line indicates a -log_10_(adjusted *P* value) of 1.3 (equals an adjusted *P* value of 0.05). The *P* value used in
-log_10_(*P* value) is the adjusted *P* value according to Benjamini Hochberg.

**Fig. S11. Detailed proteomics analysis of twenty most up- and down regulated proteins in prey populations evolved under high predatory pressure.** Heatmap of log_2_(fold change, FC) for all proteins that were detected with at least two peptides in the proteome data set. Significant values of log_2_(FC) are shown numerically (adjusted *P* value below 0.05), non-significant values are only shown by coloring. **a**, Twenty most down regulated proteins of H1-H8 overall, apart from OmpF. **b**, Twenty most up regulated proteins of H1-H8 overall. Abbreviation: NA, not detected.

**Fig. S12. Phase contrast microscopy revealed** **no differences in cell length and width between the ancestor and evolved prey population lineages, except for H8. Cell morphology changes in H8 are likely due to changes in *truA* (longer and slimmer) . a & d,** Phase contrast microscopy images of ancestor *E. coli* K-12 (AN) and evolved *E. coli* K-12 prey population lineages evolved under high predatory pressure Nr. 8 (H8, a), as well as *E. coli* BW25113 (BW, KEIO reference strain) and clean-deletion mutants Δ*envZ* and Δ*truA* thereof (**d**). The scale bar is 2 µm. **b-c & e-f**, Violin plots show the quantitative analysis of microscopy images at single cell level for width (**b**) and length (**c**) of AN and eight independently evolved *E. coli* K-12 prey population lineages that evolved under high (H1-8), low (L1-8) and no (N1-8) predatory pressure for 15 evolution cycles. Same data for width (e) and length (f) for BW, Δ*envZ* and Δ*truA*. Numbers in the panels b-c & e-f indicate the mean and standard deviation for a particular population, black dot represent median of each population and dotted line represent the median of AN or BW. The following numbers of cells per lineage/ancestor (AN) were analysed: AN: 1516, H1: 735, H2: 684, H3: 596, H4: 1350, H5: 555, H6:483 , H7: 398, H8: 434, L1: 497, L2: 422, L3: 446 ,L4: 306, L5: 514, L6: 489, L7: 368, L8: 440, N1: 1565, N2: 877, N3: 467, N4: 399, N5: 588, N6: 437, N7: 582, N8: 397, BW: 869, Δ*envZ*: 872*,* Δ*truA*: 862. Mean, median and standard deviation of single cells for **b-c & e-f** is listed in additional online material statistics table.

**Fig. S13.** **Phase contrast microscopy images of *E. coli* K-12 populations evolved under different predatory pressure for 15 evolution cycles show no morphological changes, except for line H8.** Ancestor strain *E. coli* K-12 MG1655 (AN) and eight independently evolved lines under high (H1-8), low (L1-8) and no predatory pressure (N1-8) are shown. One representative image per evolved line was selected, while for AN two different ones are shown. One image of AN and H8 are also shown in Supplementary Fig S12. Clustering of some cells on these images are very likely an artefact of the sample preparation on the slides, as two different images of AN shown. No systematic difference in dispersion was seen for all the strains except H8 with the longer chains or two cells connected ends-on. The scale bar is 2 µm.

**Fig. S14.** **Overview of experimental setup of prey evolution** as shown in Fig. 1a with added roman numbers for individual steps described in the Supplementary Methods and Materials.

**Table S1.** *E. coli* strains used and generated in this study.

| **Strain** | **Genotype** | **Source** | **Reference** |
| --- | --- | --- | --- |
| *E. coli* K-12 MG1655 | wild type  F^-^, λ^-^, *rph-*1. F*nr*+ | DSMZ (DSM 18039) | Blattner *et al.*[8] |
| *E. coli* K-12 BW25113 | wild type  *rrnB3,* ∆*lacZ4787,* *hsdR514,* ∆*(araBAD)567,* ∆*(rhaBAD)568, rph*-1 | Hall Lab, ETH Zurich | Baba *et al.*[2] |
| *E. coli* K-12 BW25113 *ompF*::*kanR* | Isogenic *ompF*::*kanR* | Hall Lab, ETHZ | Baba *et al.*[2] |
| *E. coli* K-12 BW25113 *waaF*::*kanR* | Isogenic *waaF*::*kanR* | Hall Lab, ETHZ | Baba *et al.*[2] |
| *E. coli* K-12 BW25113 *waaB*::*kanR* | Isogenic *waaB*::*kanR* | Hall Lab, ETHZ | Baba *et al.*[2] |
| *E. coli* K-12 BW2511 *envZ*::*kanR* | Isogenic *envZ*::*kanR* | Hall Lab, ETHZ | Baba *et al.*[2] |
| *E. coli* K-12 BW25113 *truA*::*kanR* | Isogenic *truA*::*kanR* | Hall Lab, ETHZ | Baba *et al.*[2] |
| *E. coli* K-12 BW25113 Δ*ompF* | Isogenic Δ*ompF* | This study |  |
| *E. coli* K-12 BW25113 Δ*waaF* (*rfaF*) | Isogenic Δ*waaF* | This study |  |
| *E. coli* K-12 BW25113 Δ*waaB* (*rfaB*) | Isogenic ΔwaaB | This study |  |
| *E. coli* K-12 BW25113 Δ*envZ* | Isogenic Δ*envZ* | This study |  |
| *E. coli* K-12 BW25113 Δ*truA* | Isogenic Δ*truA* | This study |  |

**Table S2.** Primers used to confirm clean deletion of *E. coli* BW25113 based on KEIO knock out library [2].

| **strain** | **Primer name** | **Sequence (5’ → 3’)** |
| --- | --- | --- |
| ∆*ompF* | ompF_Seq_up | gaatggaaagatgcctgc |
|  | ompF_Seq_dn | gttacagaagggaagtcc |
| ∆*waaF* | waaF_Seq_up | cttcaatctcggtactgg |
|  | waaF_Seq_dn | ctgcgtcatagttctctg |
| ∆*waaB* | waaB_Seq_up | gctggactcttcgtatac |
|  | waaB_Seq_dn | cataagcgatgtccagac |
| ∆*envZ* | envZ_Seq_up | cgtggtcgtgaatattcc |
|  | envZ_Seq_dn | ctctgccgatgtttaacc |
| ∆*truA* | truA_Seq_up | ggtattctacggtcatgc |
|  | truA_Seq_dn | taaatcaggtccatatttgc |

**Table S3.** **Conversion factor of OD_600nm_ to CFU/ml.** Determined by calculating the mean OD_600nm_ and CFU/ml counts of two no-, two low- and three high-predatory pressure evolved prey lineages at pre and post predation phases of the 15^th^ evolution cycle. These conversion factors were used to calculate the mutation supply in *ompF* gene in Supplementary Table S4.

| Predation exposure | Stage | Mean OD_600nm_ | Mean CFU/ml | OD to CFU/ml conversion factor |
| --- | --- | --- | --- | --- |
| high (H) | post predation | 0.496 | 6.45E+08 | 1.30E+09 |
| high (H) | pre predation | 1.205 | 3.18E+09 | 2.64E+09 |
| low (L) | post predation | 0.400 | 6.44E+08 | 1.61E+09 |
| low (L) | pre predation | 1.265 | 2.09E+09 | 1.65E+09 |
| no (N) | post predation | 1.064 | 5.72E+09 | 5.37E+09 |
| no (N) | pre predation | 1.303 | 3.22E+09 | 2.47E+09 |

**Table S4. Estimated number of mutations in *ompF* gene (1089 bp) after the first 24-hour predation cycle of the evolution experiment.** The total amount of prey cells based on CFU per lineage at the start was estimated to be 1’209’375 and prey mutation rate 6.24 x 10^-10^ mutations/bp [7]. For procedure see Supporting Information text, Sheet F for OD_600nm_ (OD_600nm__pred_t24 for OD_600nm_ after the first predation cycle) and Sheet_Y in xls file of source data.

| **Lineage** | **Prey cells at end of 1st predation cycle** | **Generations** | **Generation time (in h)** | **Number in mutations in *ompF*** |
| --- | --- | --- | --- | --- |
| **L1** | 920’900’551 | 9.6 | 2.5 | 626 |
| **L2** | 930’542’246 | 9.6 | 2.5 | 632 |
| **L3** | 915’356’530 | 9.6 | 2.5 | 622 |
| **L4** | 913’428’306 | 9.6 | 2.5 | 621 |
| **L5** | 961’516’078 | 9.6 | 2.5 | 653 |
| **L6** | 952’597’485 | 9.6 | 2.5 | 647 |
| **L7** | 979’835’283 | 9.7 | 2.5 | 666 |
| **L8** | 860’760’586 | 9.5 | 2.5 | 585 |
| **H1** | 370’938’624 | 8.3 | 2.9 | 252 |
| **H2** | 143’306’949 | 6.9 | 3.5 | 97 |
| **H3** | 71’157’439 | 5.9 | 4.1 | 48 |
| **H4** | 22’997’654 | 4.2 | 5.6 | 16 |
| **H5** | 345’235’333 | 8.2 | 2.9 | 235 |
| **H6** | 98’844’821 | 6.4 | 3.8 | 67 |
| **H7** | 72’149’511 | 5.9 | 4.1 | 49 |
| **H8** | 11’002’798 | 3.2 | 7.5 | 7 |

**Table S5. Table of gene mutations in evolved prey populations.** Detailed characterisation of all mutations (>5% difference from the *E. coli* K-12 ancestor) within a coding gene in prey lineages H1-8, L1-L8) exclusively evolved under high and/or low predatory pressure (see Fig. 4). The same colour code was used as in Fig. 4 for the mutation type. Abbreviations: SNP: Single nucleotide polymorphism, InDel: Insertion or Deletion, fs: frame shift, *: early stop codon gained.

| **gene** | **prey** | **%** | **impact** | **type** | **mutation type** | **change** |
| --- | --- | --- | --- | --- | --- | --- |
| *ompF* | H2 | 99.6 | moderate | SNP | missense variant | Ser294Pro |
| *ompF* | H4 | 99.9 | high | InDel | frameshift | Tyr332fs |
| *ompF* | H6 | 96.9 | high | InDel | frameshift | Glu70fs |
| *ompF* | H7 | 73.6 | high | InDel | frameshift | Asn183fs |
| *ompF* | H7 | 25.7 | high | InDel | frameshift | Tyr332fs |
| *ompF* | L1 | 100 | high | SNP | stop gained | Gln88* |
| *ompF* | L2 | 5.1 | high | SNP | stop gained | Gln88* |
| *waaF* | H5 | 100 | moderate | SNP | missense variant | Ala255Glu |
| *envZ* | H8 | 77.5 | moderate | InDel | conservative inframe insertion | Insertion of Lys-Thr-Trp-Leu between Leu135 and Lys136 |
| *truA* | H8 | 100 | moderate | SNP | missense variant | Val200Ala |
| *waaB* | H4 | 87.5 | high | SNP | stop gained | Glu17* |
| *ddpX* | H7 | 28.3 | low | SNP | synonymous variant | Val92Val (codon usage decreased) |
| *yhfA* | H7 | 18.1 | low | SNP | synonymous variant | Leu96Leu (codon usage decreased) |
| *sseA* | H1 | -6.2 | moderate | SNP | missense variant | Lys214Asn |
| *sseA* | H5 | -5.7 | moderate | SNP | missense variant | Lys214Asn |
| *sseA* | L2 | -5.2 | moderate | SNP | missense variant | Lys214Asn |
| *sseA* | L6 | -5.4 | moderate | SNP | missense variant | Lys214Asn |
| *yfhM* | H5 | 5.2 | low | SNP | synonymous variant | Val712Val (codon usage invariant) |
| *fimD* | H5 | 7.9 | low | SNP | synonymous variant | Pro838Pro (codon usage decreased) |
| *puuC* | H5 | 7.2 | moderate | SNP | missense variant | Asp388Glu |
| *lolD* | H5 | 10.0 | low | SNP | synonymous variant | His141His (codon usage optimised) |
| *lsrD* | H5 | 12.7 | low | SNP | synonymous variant | Gly187Gly (codon usage optimised) |
| *argD* | H2 | 7.4 | low | SNP | synonymous variant | Leu257Leu (codon usage decreased) |
| *ppiA* | H2 | 6.4 | high | InDel | frameshift | Thr85fs |
| *ppiA* | H2 | 9.5 | moderate | SNP | missense variant | Thr85Pro |
| *gcvP* | H3 | 9.3 | moderate | SNP | missense variant | Tyr109Ser |
| *ybgS* | H3 | -5.4 | moderate | SNP | missense variant | Thr121Pro |
| *mlaF* | H4 | 8.7 | high | SNP | stop gained | Gln175* |
| *oppB* | H4 | 5.3 | moderate | SNP | missense variant | Phe75Leu |
| *chbC* | H1 | -5.4 | moderate | SNP | missense variant | Phe60Cys |
| *chbC* | L4 | -5.3 | moderate | SNP | missense variant | Phe60Cys |
| *ypdE* | L4 | 8.0 | low | SNP | synonymous variant | Ala303Ala (codon usage optimised) |
| *ydjI* | L8 | 5.4 | moderate | SNP | missense variant | Asn139Lys |
| *yfbN* | L8 | 7.8 | moderate | SNP | missense variant | Ile18Ser |
| *yhjJ* | L3 | 5.9 | moderate | SNP | missense variant | Ile115Met |
| *yhjJ* | L3 | 5.2 | moderate | SNP | missense variant | Ile115Ser |
| *pdeR* | H7 | 5.3 | moderate | SNP | missense variant | Ala543Val |
| *yadL* | L1 | 5.6 | low | SNP | synonymous variant | Val166Val (codon usage optimised) |

**Table S6.** (in separate xls-table online) **Table containing all significantly regulated proteins between evolved lineages (H1-H8) and ancestor, as well as Δ*ompF* and Δ*waaF* to the KEIO reference strain *E. coli* K-12 BW25113.** For each comparison it is listed: UniProt protein accession number, associated gene names, description, Log_2_(fold change, FC), -Log_10_(FDR), adjusted *P* value according to Benjamini Hochberg) (FDR). The “status” indicates significant (FDR < 0.05) up or down regulation. The different comparisons are separated by different tabs.

**Table S7.** Peptides detected of OmpF in the different *E. coli* lineages by mass spectrometry in one (yellow), two (light orange) or three (dark orange) out of three biological replicates. If peptides were overlapping, then the colour of the one showing the higher replicate number was retained. Mutations within *ompF* were added after the lineage name for a better overview (fs = frame shift).

| **Lineage** | **OmpF sequence with MS-identified peptides in grey** |
| --- | --- |
| K-12 MG1655 ancestor | MMKRNILAVIVPALLVAGTANAAEIYNKDGNKVDLYGKAVGLHYFSKGNGENSYGGNGDMTYARLGFKGETQINSDLTGYGQWEYNFQGNNSEGADAQTGNKTRLAFAGLKYADVGSFDYGRNYGVVYDALGYTDMLPEFGGDTAYSDDFFVGRVGGVATYRNSNFFGLVDGLNFAVQYLGKNERDTARRSNGDGVGGSISYEYEGFGIVGAYGAADRTNLQEAQPLGNGKKAEQWATGLKYDANNIYLAANYGETRNATPITNKFTNTSGFANKTQDVLLVAQYQFDFGLRPSIAYTKSKAKDVEGIGDVDLVNYFEVGATYYFNKNMSTYVDYIINQIDSDNKLGVGSDDTVAVGIVYQF |
| H1 | MMKRNILAVIVPALLVAGTANAAEIYNKDGNKVDLYGKAVGLHYFSKGNGENSYGGNGDMTYARLGFKGETQINSDLTGYGQWEYNFQGNNSEGADAQTGNKTRLAFAGLKYADVGSFDYGRNYGVVYDALGYTDMLPEFGGDTAYSDDFFVGRVGGVATYRNSNFFGLVDGLNFAVQYLGKNERDTARRSNGDGVGGSISYEYEGFGIVGAYGAADRTNLQEAQPLGNGKKAEQWATGLKYDANNIYLAANYGETRNATPITNKFTNTSGFANKTQDVLLVAQYQFDFGLRPSIAYTKSKAKDVEGIGDVDLVNYFEVGATYYFNKNMSTYVDYIINQIDSDNKLGVGSDDTVAVGIVYQF |
| H2 (Ser294Pro) | MMKRNILAVIVPALLVAGTANAAEIYNKDGNKVDLYGKAVGLHYFSKGNGENSYGGNGDMTYARLGFKGETQINSDLTGYGQWEYNFQGNNSEGADAQTGNKTRLAFAGLKYADVGSFDYGRNYGVVYDALGYTDMLPEFGGDTAYSDDFFVGRVGGVATYRNSNFFGLVDGLNFAVQYLGKNERDTARRSNGDGVGGSISYEYEGFGIVGAYGAADRTNLQEAQPLGNGKKAEQWATGLKYDANNIYLAANYGETRNATPITNKFTNTSGFANKTQDVLLVAQYQFDFGLRPSIAYTKSKAKDVEGIGDVDLVNYFEVGATYYFNKNMSTYVDYIINQIDSDNKLGVGSDDTVAVGIVYQF |
| H3 | MMKRNILAVIVPALLVAGTANAAEIYNKDGNKVDLYGKAVGLHYFSKGNGENSYGGNGDMTYARLGFKGETQINSDLTGYGQWEYNFQGNNSEGADAQTGNKTRLAFAGLKYADVGSFDYGRNYGVVYDALGYTDMLPEFGGDTAYSDDFFVGRVGGVATYRNSNFFGLVDGLNFAVQYLGKNERDTARRSNGDGVGGSISYEYEGFGIVGAYGAADRTNLQEAQPLGNGKKAEQWATGLKYDANNIYLAANYGETRNATPITNKFTNTSGFANKTQDVLLVAQYQFDFGLRPSIAYTKSKAKDVEGIGDVDLVNYFEVGATYYFNKNMSTYVDYIINQIDSDNKLGVGSDDTVAVGIVYQF |
| H4 (Tyr332fs) | MMKRNILAVIVPALLVAGTANAAEIYNKDGNKVDLYGKAVGLHYFSKGNGENSYGGNGDMTYARLGFKGETQINSDLTGYGQWEYNFQGNNSEGADAQTGNKTRLAFAGLKYADVGSFDYGRNYGVVYDALGYTDMLPEFGGDTAYSDDFFVGRVGGVATYRNSNFFGLVDGLNFAVQYLGKNERDTARRSNGDGVGGSISYEYEGFGIVGAYGAADRTNLQEAQPLGNGKKAEQWATGLKYDANNIYLAANYGETRNATPITNKFTNTSGFANKTQDVLLVAQYQFDFGLRPSIAYTKSKAKDVEGIGDVDLVNYFEVGATYYFNKNMSTYVDYIINQIDSDNKLGVGSDDTVAVGIVYQF |
| H5 | MMKRNILAVIVPALLVAGTANAAEIYNKDGNKVDLYGKAVGLHYFSKGNGENSYGGNGDMTYARLGFKGETQINSDLTGYGQWEYNFQGNNSEGADAQTGNKTRLAFAGLKYADVGSFDYGRNYGVVYDALGYTDMLPEFGGDTAYSDDFFVGRVGGVATYRNSNFFGLVDGLNFAVQYLGKNERDTARRSNGDGVGGSISYEYEGFGIVGAYGAADRTNLQEAQPLGNGKKAEQWATGLKYDANNIYLAANYGETRNATPITNKFTNTSGFANKTQDVLLVAQYQFDFGLRPSIAYTKSKAKDVEGIGDVDLVNYFEVGATYYFNKNMSTYVDYIINQIDSDNKLGVGSDDTVAVGIVYQF |
| H6 (Glu70fs) | MMKRNILAVIVPALLVAGTANAAEIYNKDGNKVDLYGKAVGLHYFSKGNGENSYGGNGDMTYARLGFKGETQINSDLTGYGQWEYNFQGNNSEGADAQTGNKTRLAFAGLKYADVGSFDYGRNYGVVYDALGYTDMLPEFGGDTAYSDDFFVGRVGGVATYRNSNFFGLVDGLNFAVQYLGKNERDTARRSNGDGVGGSISYEYEGFGIVGAYGAADRTNLQEAQPLGNGKKAEQWATGLKYDANNIYLAANYGETRNATPITNKFTNTSGFANKTQDVLLVAQYQFDFGLRPSIAYTKSKAKDVEGIGDVDLVNYFEVGATYYFNKNMSTYVDYIINQIDSDNKLGVGSDDTVAVGIVYQF |
| H7 (Asn183fs) or (Tyr332fs) | MMKRNILAVIVPALLVAGTANAAEIYNKDGNKVDLYGKAVGLHYFSKGNGENSYGGNGDMTYARLGFKGETQINSDLTGYGQWEYNFQGNNSEGADAQTGNKTRLAFAGLKYADVGSFDYGRNYGVVYDALGYTDMLPEFGGDTAYSDDFFVGRVGGVATYRNSNFFGLVDGLNFAVQYLGKNERDTARRSNGDGVGGSISYEYEGFGIVGAYGAADRTNLQEAQPLGNGKKAEQWATGLKYDANNIYLAANYGETRNATPITNKFTNTSGFANKTQDVLLVAQYQFDFGLRPSIAYTKSKAKDVEGIGDVDLVNYFEVGATYYFNKNMSTYVDYIINQIDSDNKLGVGSDDTVAVGIVYQF |
| H8 | MMKRNILAVIVPALLVAGTANAAEIYNKDGNKVDLYGKAVGLHYFSKGNGENSYGGNGDMTYARLGFKGETQINSDLTGYGQWEYNFQGNNSEGADAQTGNKTRLAFAGLKYADVGSFDYGRNYGVVYDALGYTDMLPEFGGDTAYSDDFFVGRVGGVATYRNSNFFGLVDGLNFAVQYLGKNERDTARRSNGDGVGGSISYEYEGFGIVGAYGAADRTNLQEAQPLGNGKKAEQWATGLKYDANNIYLAANYGETRNATPITNKFTNTSGFANKTQDVLLVAQYQFDFGLRPSIAYTKSKAKDVEGIGDVDLVNYFEVGATYYFNKNMSTYVDYIINQIDSDNKLGVGSDDTVAVGIVYQF |
| BW25113 | MMKRNILAVIVPALLVAGTANAAEIYNKDGNKVDLYGKAVGLHYFSKGNGENSYGGNGDMTYARLGFKGETQINSDLTGYGQWEYNFQGNNSEGADAQTGNKTRLAFAGLKYADVGSFDYGRNYGVVYDALGYTDMLPEFGGDTAYSDDFFVGRVGGVATYRNSNFFGLVDGLNFAVQYLGKNERDTARRSNGDGVGGSISYEYEGFGIVGAYGAADRTNLQEAQPLGNGKKAEQWATGLKYDANNIYLAANYGETRNATPITNKFTNTSGFANKTQDVLLVAQYQFDFGLRPSIAYTKSKAKDVEGIGDVDLVNYFEVGATYYFNKNMSTYVDYIINQIDSDNKLGVGSDDTVAVGIVYQF |
| ∆*ompF* | MMKRNILAVIVPALLVAGTANAAEIYNKDGNKVDLYGKAVGLHYFSKGNGENSYGGNGDMTYARLGFKGETQINSDLTGYGQWEYNFQGNNSEGADAQTGNKTRLAFAGLKYADVGSFDYGRNYGVVYDALGYTDMLPEFGGDTAYSDDFFVGRVGGVATYRNSNFFGLVDGLNFAVQYLGKNERDTARRSNGDGVGGSISYEYEGFGIVGAYGAADRTNLQEAQPLGNGKKAEQWATGLKYDANNIYLAANYGETRNATPITNKFTNTSGFANKTQDVLLVAQYQFDFGLRPSIAYTKSKAKDVEGIGDVDLVNYFEVGATYYFNKNMSTYVDYIINQIDSDNKLGVGSDDTVAVGIVYQF |
| ∆*waaF* | MMKRNILAVIVPALLVAGTANAAEIYNKDGNKVDLYGKAVGLHYFSKGNGENSYGGNGDMTYARLGFKGETQINSDLTGYGQWEYNFQGNNSEGADAQTGNKTRLAFAGLKYADVGSFDYGRNYGVVYDALGYTDMLPEFGGDTAYSDDFFVGRVGGVATYRNSNFFGLVDGLNFAVQYLGKNERDTARRSNGDGVGGSISYEYEGFGIVGAYGAADRTNLQEAQPLGNGKKAEQWATGLKYDANNIYLAANYGETRNATPITNKFTNTSGFANKTQDVLLVAQYQFDFGLRPSIAYTKSKAKDVEGIGDVDLVNYFEVGATYYFNKNMSTYVDYIINQIDSDNKLGVGSDDTVAVGIVYQF |

**SI References**

1.	Lambert C, Sockett RE. Laboratory maintenance of Bdellovibrio. *Curr Protoc Microbiol* 2008;**Chapter 7**:Unit 7B.2. https://doi.org/10.1002/9780471729259.mc07b02s9

2.	Baba T et al. Construction of *Escherichia coli* K‐12 in‐frame, single‐gene knockout mutants: the Keio collection. *Molecular Systems Biology* 2006;**2**:2006.0008. https://doi.org/10.1038/msb4100050

3.	Datsenko KA, Wanner BL. One-step inactivation of chromosomal genes in *Escherichia coli* K-12 using PCR products. *Proc Natl Acad Sci USA* 2000;**97**:6640–6645. https://doi.org/10.1073/pnas.120163297

4.	Barrick Lab. Barrick Lab :: ProtocolsElectrocompetentCells. https://barricklab.org/twiki/bin/view/Lab/ProtocolsElectrocompetentCells. .

5.	Barrick Lab. Barrick Lab :: ProcedureFLPFRTRecombination. https://barricklab.org/twiki/bin/view/Lab/ProcedureFLPFRTRecombination. .

6.	Vollenweider V, Roncoroni F, Kümmerli R. Pyoverdine–antibiotic combination treatment: its efficacy and effects on resistance evolution in *Escherichia coli*. *microLife* 2024;**5**:uqae021. https://doi.org/10.1093/femsml/uqae021

7.	McElroy KE et al. Strain-specific parallel evolution drives short-term diversification during *Pseudomonas aeruginosa* biofilm formation. *Proc Natl Acad Sci USA* 2014;**111**. https://doi.org/10.1073/pnas.1314340111

8.	Blattner FR et al. The Complete Genome Sequence of *Escherichia coli* K-12. *Science* 1997;**277**:1453–1462. https://doi.org/10.1126/science.277.5331.1453

9.	Hayashi K et al. Highly accurate genome sequences of *Escherichia coli* K‐12 strains MG1655 and W3110. *Molecular Systems Biology* 2006;**2**:2006.0007. https://doi.org/10.1038/msb4100049

10.	Kolde, R. Pheatmap: pretty heatmaps. R Package Version 1.0.10., 2019.

11.	Chen, H. VennDiagram: Generate High-Resolution Venn and Euler Plots. R package version 1.7.3, 2022.

12.	Bolger AM, Lohse M, Usadel B. Trimmomatic: a flexible trimmer for Illumina sequence data. *Bioinformatics* 2014;**30**:2114–2120. https://doi.org/10.1093/bioinformatics/btu170

13.	Bankevich A et al. SPAdes: A New Genome Assembly Algorithm and Its Applications to Single-Cell Sequencing. *Journal of Computational Biology* 2012;**19**:455–477. https://doi.org/10.1089/cmb.2012.0021

14.	Seemann T. Prokka: rapid prokaryotic genome annotation. *Bioinformatics* 2014;**30**:2068–2069. https://doi.org/10.1093/bioinformatics/btu153

15.	Li H. Aligning sequence reads, clone sequences and assembly contigs with BWA-MEM. 2013.

16.	Danecek P et al. Twelve years of SAMtools and BCFtools. *GigaScience* 2021;**10**:giab008. https://doi.org/10.1093/gigascience/giab008

17.	Koboldt DC et al. VarScan: variant detection in massively parallel sequencing of individual and pooled samples. *Bioinformatics* 2009;**25**:2283–2285. https://doi.org/10.1093/bioinformatics/btp373

18.	Cingolani P et al. A program for annotating and predicting the effects of single nucleotide polymorphisms, SnpEff: SNPs in the genome of Drosophila melanogaster strain w ^1118^ ; iso-2; iso-3. *Fly* 2012;**6**:80–92. https://doi.org/10.4161/fly.19695

19.	Türker C et al. B-Fabric: the Swiss Army Knife for life sciences. *Proceedings of the 13th International Conference on Extending Database Technology*. 2010. Lausanne Switzerland: ACM, 2010, pp 717–720.

20.	Wolski WE et al. *prolfqua* : A Comprehensive *R* -Package for Proteomics Differential Expression Analysis. *J Proteome Res* 2023;**22**:1092–1104. https://doi.org/10.1021/acs.jproteome.2c00441

21.	Huber W et al. Variance stabilization applied to microarray data calibration and to the quantification of differential expression. *Bioinformatics* 2002;**18**:S96–S104. https://doi.org/10.1093/bioinformatics/18.suppl_1.S96

22.	Leary P, Rehrauer H. exploreDE Interactive Shiny App. 2024. Zenodo, 2024.

23.	R Core Team. R: A language and environment for statistical computing. Vienna, Austria: Vienna, Austria, 2021.

24.	Wickham H et al. Welcome to the Tidyverse. *JOSS* 2019;**4**:1686. https://doi.org/10.21105/joss.01686

25.	Wickham H. ggplot2: Elegant Graphics for Data Analysis, 2016th edn. Springer-Verlag New York, 2016.

26.	Mirdita M et al. ColabFold: making protein folding accessible to all. *Nat Methods* 2022;**19**:679–682. https://doi.org/10.1038/s41592-022-01488-1

27.	Kim G et al. Easy and accurate protein structure prediction using ColabFold. *Nat Protoc* 2025;**20**:620–642. https://doi.org/10.1038/s41596-024-01060-5

28.	Yamashita E et al. Crystal structures of the OmpF porin: function in a colicin translocon. *EMBO J* 2008;**27**:2171–2180. https://doi.org/10.1038/emboj.2008.137
